# Surface-Assisted Selective Air Oxidation of Phosphines Adsorbed on Activated Carbon

**DOI:** 10.1021/acs.inorgchem.4c01027

**Published:** 2024-05-09

**Authors:** John C. Hoefler, Devin Jackson, Janet Blümel

**Affiliations:** Department of Chemistry, Texas A&M University, College Station, Texas 77845-3012, United States

## Abstract

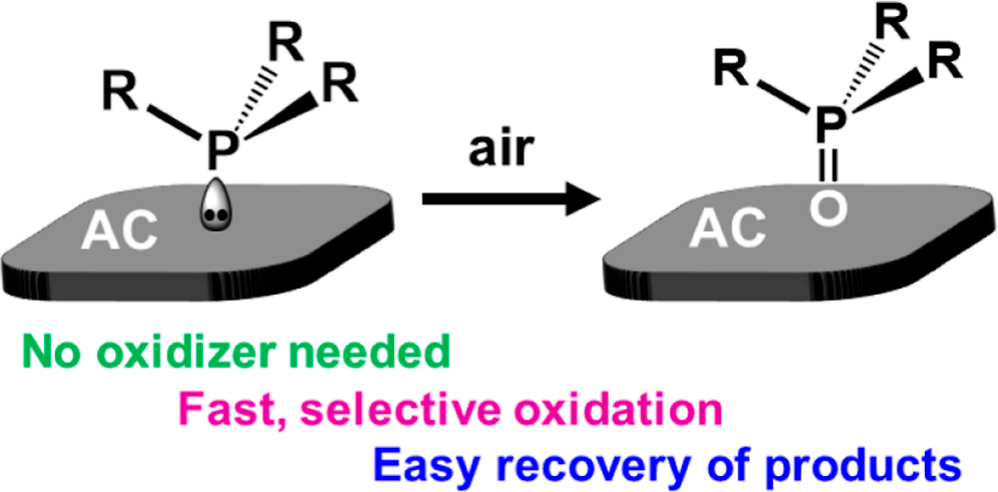

Trialkyl- and triarylphosphines readily adsorb onto the surface of porous activated carbon (AC) even in the absence of solvents through van der Waals interactions between the lone electron pair and the AC surface. This process has been proven by solid-state NMR techniques. Subsequently, it is demonstrated that the AC enables the fast and selective oxidation of adsorbed phosphines to phosphine oxides at ambient temperature in air. In solution, trialkylphosphines are oxidized to a variety of P(V) species when exposed to the atmosphere, while neat or dissolved triarylphosphines cannot be oxidized with air. When the trialkyl- and triarylphosphines P^*n*^Bu_3_ (**1**), PEt_3_, (**2**), P^*n*^Oct_3_ (**3**), PMe^*t*^Bu_2_ (**4**), PCy_3_ (**5**), and PPh_3_ (**6**) are adsorbed in a mono- or submonolayer on the surface of AC, in the absence of a solvent and at ambient temperature, they are quantitatively oxidized to the adsorbed phosphine oxides, **1**_**ox**_–**6**_**ox**_, once air is admitted. No formation of any unwanted P(V) side products or water adducts is observed. The phosphine oxides can then be recovered in good yields by washing them off of the AC. The oxidation is likely facilitated by a radical activation of molecular oxygen due to delocalized electrons on the aromatic surface coating of AC, as proven by ESR. This easy and inexpensive oxidation method renders hydrogen peroxide or other oxidizers unnecessary and is broadly applicable to sterically hindered and even to air-stable triarylphosphines. Phosphines adsorbed at lower surface coverages on AC oxidize at a faster rate. All oxidation reactions were monitored by solution- and solid-state NMR spectroscopy.

## Abstract 2

Trialkyl- and triarylphosphines readily adsorb onto the surface of porous activated carbon (AC) even in the absence of solvents through van der Waals interactions between the lone electron pair and the AC surface.

## 1 Introduction

Adsorption of molecules on surfaces is a crucially important process with wide-ranging applications in academia and industry. Therefore, new developments in the separation sciences, as well as physical and analytical chemistry, appear frequently in the chemical literature.^1−6^ Volatile adsorbates can be removed in vacuo, while washing with favorable solvents removes nonvolatile adsorbates.

One class of molecules whose adsorption has been studied by our group and others is phosphines.^7−10^ Owing to the lone electron pair of the P atom, phosphines adsorb, for example, onto porous metal oxide surfaces such as silica via van der Waals interactions.^10^ The adsorbed phosphines are highly mobile on the surface, and the adsorption is reversible. This phenomenon has been studied using multinuclear solid-state NMR spectroscopy, which is a powerful technique for surface species and materials characterization.^10−17^

Phosphines are ubiquitous in chemistry due to their ability to coordinate to metal centers. Most recently, it was discovered that simple phosphines can even be applied as catalysts.^18^ In contrast, phosphine oxides were regarded more as an unwanted impurity in phosphorus chemistry for a long time. However, nowadays phosphine oxides enjoy growing interest. Their ability to form adducts with amines and Lewis acids renders them useful crystallization aids,^19,20^ and they function as intermediates^21^ or synthetic targets.^22−24^ Phosphine oxides are important for Mitsunobu reactions,^25,26^ and recently, redox-free Mitsunobu organocatalysis has been reported.^27^ Furthermore, phosphine oxides have been used to investigate surface acidities,^28,29^ and they play an important role in decomposing chemical warfare agents.^30^

Importantly, as electron pair donors, phosphine oxides can form hydrogen bonds with a variety of species containing OH groups.^31−38^ For example, phosphine oxides can create networks with phenols,^31^ and they engage in hydrogen bonding with water^35−37^ and silanols.^38^ Phosphine oxides also have the ability to stabilize hydrogen peroxide and di(hydroperoxy)alkanes in the form of Hilliard adducts,^36,37^ (R_3_PO·H_2_O_2_)_2_, and Ahn adducts,^39−45^ R_3_PO·(HOO)_2_CR’R″ (R,R′,R″ = alkyl, aryl). Finally, phosphine oxides interact strongly with various oxide surfaces like silica and alumina via hydrogen bonding.^46−48^

**Figure 1:**
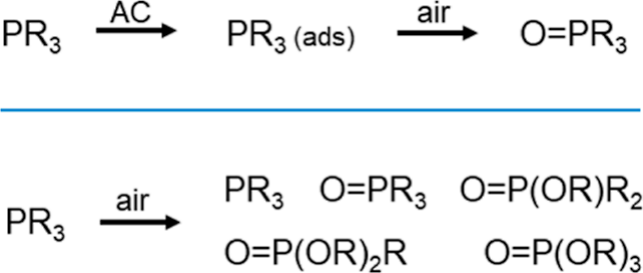
When dissolved or neat phosphines are oxidized in air, a variety of side products are obtained (bottom).^36,49^ Adsorbing the phosphines first on activated carbon (AC) leads to selective oxidation in air, and the clean phosphine oxides can be washed off of the AC surface (top).

Despite the growing interest in phosphine oxide chemistry, their synthesis from phosphines is not trivial. While most trialkylphosphines (PR_3_) are very air sensitive, they are not oxidized cleanly when exposed to the atmosphere. In addition to the desired oxide (O=PR_3_), other P(V) species are generated (Figure 1).^36,49−52^ This has been proposed to occur due to the formation of RO_2_^•^ radicals from PR_3_ which then react with other PR_3_ molecules to either form the desired products O=PR_3_ and RO^•^ or the unwanted phosphinic acid ester OP(OR)R_2_ and O^•^.^52^ Triarylphosphines, on the other hand, are often resistant to air oxidation, and the reasons have been investigated by Barder and Buchwald.^49^ In fact, at present, the best method to create clean phosphine oxides is the oxidation of phosphines with hydrogen peroxide, followed by destruction of the formed adducts and removal of the produced water with molecular sieves.^36^ While this method is doable, it involves organic solvents in combination with the expensive and perishable hydrogen peroxide that is a potential safety hazard. Reduction of the undesired phosphonates after oxidation using strong reducing agents is also possible, albeit cumbersome.^53,54^ More recently, an electrochemical oxidation of phosphines using H_2_O has been described.^55^

Searching for an efficient air oxygen-based method for phosphine oxide synthesis remains an attractive goal, and fortunately, we found a new convenient route to selectively oxidize phosphines to the corresponding phosphine oxides. Given the known affinity of phosphines to adsorb on silica,^10^ and our previous experience with metallocenes adsorbed on activated carbon (AC),^56,57^ we sought to study trialkyl- and triarylphosphines adsorbed on AC. However, we soon discovered that PPh_3_, which is not air sensitive in neat form or dissolved, is readily and selectively oxidized to O=PR_3_ when adsorbed on AC and exposed to the atmosphere. Intrigued by this phenomenon and given our early interest in synthesizing clean phosphine oxides,^36^ we aimed to investigate the adsorption and air oxidation of phosphines on AC in more detail. We assumed that AC activates molecular O_2_ by forming surface-adsorbed radicals of O_2_, in this way limiting the formation of RO_2_^•^ radicals^52^ from adsorbed phosphines and favoring the clean oxidation pathway outlined at the top of Figure 1. A more detailed analysis is provided at a later point in this contribution.

The AC surface is less well defined than silica surfaces, and various brands of AC are mostly employed for the removal of volatile organic compounds (VOCs) from the atmosphere.^58,59^ The syntheses of different AC brands depend on their intended applications. For example, during the production of AC, additives such as acids or metals can be incorporated to facilitate the conversion of subsequently adsorbed molecules.^60,61^ Such techniques have been applied for the oxidation of adsorbed nitrous oxide, methyl amines, and hydrogen sulfide.^62−68^ However, the primary surface of AC consists of aromatic ring systems that contain delocalized unpaired electrons.^69,70^ The presence of radicals has allowed oxidation reactions of adsorbed substrates using only O_2_ from the air.^71,72^ These studies suggest that adsorption of O_2_ on the AC surface is facilitated by the delocalized electrons. Adsorbed O_2_ is crucial, as higher oxidation rates are observed when AC types with higher surface areas are used, but the yields are unchanged when acid treatments are applied to the AC to remove any trace metals that may be present.^71,72^ Similar results have recently been found for adsorbed substrates on SiO_2_ with an SiO^•^ radical proposed as being responsible for oxidations.^73^ However, we have never observed oxidation of triarylphosphines or tricyclohexylphosphine on any type of the SiO_2_ surface.^10^

**Figure 2:**
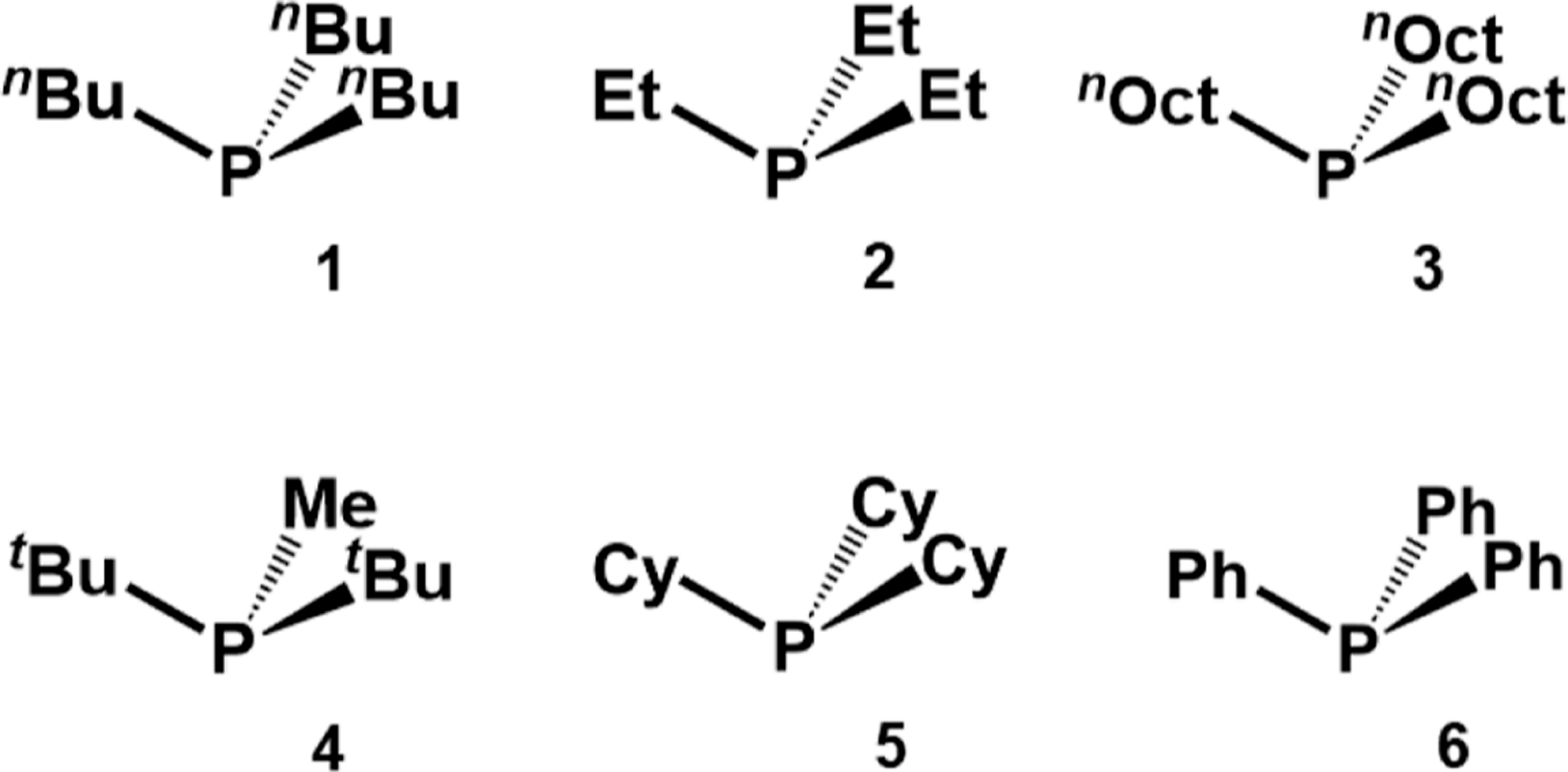
Phosphines **1**–**6** with substituents that exhibit different Tolman angles of 132° (**1** and **2**),^74^ 130° (**3**),^75^ 161° (**4**), 170° (**5**),^74^ and 145° (**6**).^74^

In the following, it will be demonstrated that tertiary alkyl- and arylphosphines can be adsorbed onto AC surfaces not only from solutions but also by mixing the neat, and in some cases solid, components, as described previously for silica surfaces.^10^ So far, phosphine adsorption studies on AC have been limited to gas-phase adsorption of volatile phosphines like PH_3_.^7^ A timeline for the PR_3_ adsorption process was established using solid-state NMR analyses. Importantly, the phosphines have to be adsorbed under an inert gas atmosphere. When oxygen is admitted, the phosphines are oxidized to the corresponding phosphine oxides. It will be demonstrated that AC-adsorbed P^*n*^Bu_3_ (**1**), PEt_3_ (**2**), P^*n*^Oct_3_ (**3**), PMe^*t*^Bu_2_ (**4**), PCy_3_ (**5**), and PPh_3_ (**6**) (Figure 2) can be oxidized selectively by air to give clean phosphine oxides **1**_**ox**_–**6**_**ox**_ (Figure 1). To achieve clean oxidation, the phosphines are first adsorbed in a mono- or submonolayer on the AC surface. The described process simplifies the synthesis of phosphine oxides substantially.

## 2 Results and Discussion

### 2.1 Adsorption and Oxidation of PPh_3_ (6) on AC

We have reported earlier that phosphines with high melting points like PCy_3_ and PPh_3_ readily adsorb on silica surfaces.^10^ The adsorption takes place within minutes even when no solvent is involved, and the dry components are mixed manually. The dynamics of the phosphine molecules on the silica surface was studied in detail.^10^ To test the general nature of the dynamic phenomena and probe the interactions with a surface different from the silica used previously, we applied activated carbon (AC) as the adsorbent. Since PPh_3_ (**6**) is solid, inexpensive, not air sensitive, and therefore easy to handle, we started adsorbing this phosphine on AC.

**Figure 3:**
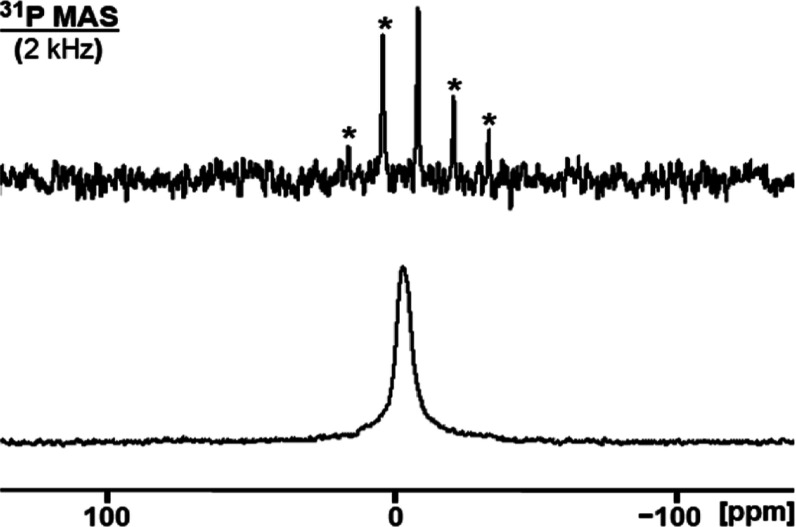
^31^P MAS NMR spectrum of polycrystalline **6** (top) and **6** adsorbed on AC with 98% surface coverage (bottom).

In order to verify that **6** was adsorbed and mobile on the AC surface, and not just residing as a polycrystalline material in the pores, we used solid-state NMR. In general, when a surface-adsorbed species is mobile on the surface, the signals of adsorbed species show significantly reduced chemical shift anisotropy (CSA)^11,12^ compared to their polycrystalline forms.^4,5,10,46−48^ The reason for this is that the CSA is an anisotropic interaction that is averaged out when a molecule is highly mobile and reorients quickly like in solution. Surface-adsorbed species in porous materials display translational mobility, spiraling on the surface within the pores and therewith they behave like in solution. At low magic angle spinning (MAS) speeds, signals with large CSA typically feature several sets of rotational sidebands that occur at intervals equal to the rotational speed. This is the case when polycrystalline **6** is measured using ^31^P MAS NMR at a slow rotational speed of 2 kHz (Figure 3).^10^

Phosphine **6** can be adsorbed on AC under an inert atmosphere by manually grinding the solid components in the absence of a solvent. Alternatively, a solution of **6** can be applied to AC, and **6** remains adsorbed when the solvent is removed in vacuo. The surface coverage of AC with **6** can be determined using the standard procedure described in the Experimental Section. After the adsorption of **6** on AC with 98% surface coverage, the ^31^P MAS NMR spectrum was recorded and compared to that of polycrystalline **6** (Figure 3). The CSA is averaged out, and no rotational sidebands remain. This indicates that the molecules of **6** are spread out on the surface and undergo rapid reorientation within the AC pores. No polycrystalline **6** is left. Characteristically, the halfwidth of the isotropic line increases from 120 to 1000 Hz upon adsorption, a trend consistent with what has been reported for **6** adsorbed on silica.^10^ Phosphines adsorbed on amorphous surfaces encounter different environments, hence the broader residual lines as compared to molecules residing in well-defined crystal lattices. Finally, there is a downfield shift of the isotropic line for adsorbed **6** to about −4.1 ppm as compared to the chemical shift of the signal of polycrystalline **6** at −9.4 ppm.^10^ The downfield shift can be attributed to the interactions of the lone electron pair of the P atom in **6** with the AC surface and, therefore, lower electron density at the P atom. This interaction and its impact on ^31^P NMR signals have previously been observed for phosphines adsorbed on silica surfaces.^10^

As additional proof for adsorption, the ^31^P T_1_ relaxation times of polycrystalline and adsorbed **6** can be employed. Polycrystalline materials have extremely long T_1_ relaxation times when measured without CP (cross-polarization).^76^ For example, ethane-1,2-diphosphonic acid features a ^31^P T_1_ relaxation time of 900 s,^77^ and ∼1000 s have been reported for polycrystalline PPh_3_.^78^ In contrast, we determined the T_1_ time for the ^31^P nucleus in adsorbed **6** as 0.16 s (Figures S1 and S2 and Tables S1 and S2). Due to the mobility of the adsorbed phosphine, the T_1_ relaxation is orders of magnitude faster than for polycrystalline PPh_3_. The T_1_ value for AC-adsorbed **6** is the same order of magnitude as that reported for PPh_3_ adsorbed on silica (0.59 s).^10^ The faster T_1_ relaxation for **6** adsorbed on AC is probably due to the paramagnetic nature of the AC surface. In summary, the removal of the CSA, the downfield shift of the isotropic ^31^P line, the increased residual line width, and the significant reduction of the ^31^P T_1_ relaxation time prove that **6** is adsorbed on the AC surface and mobile.

**Figure 4:**
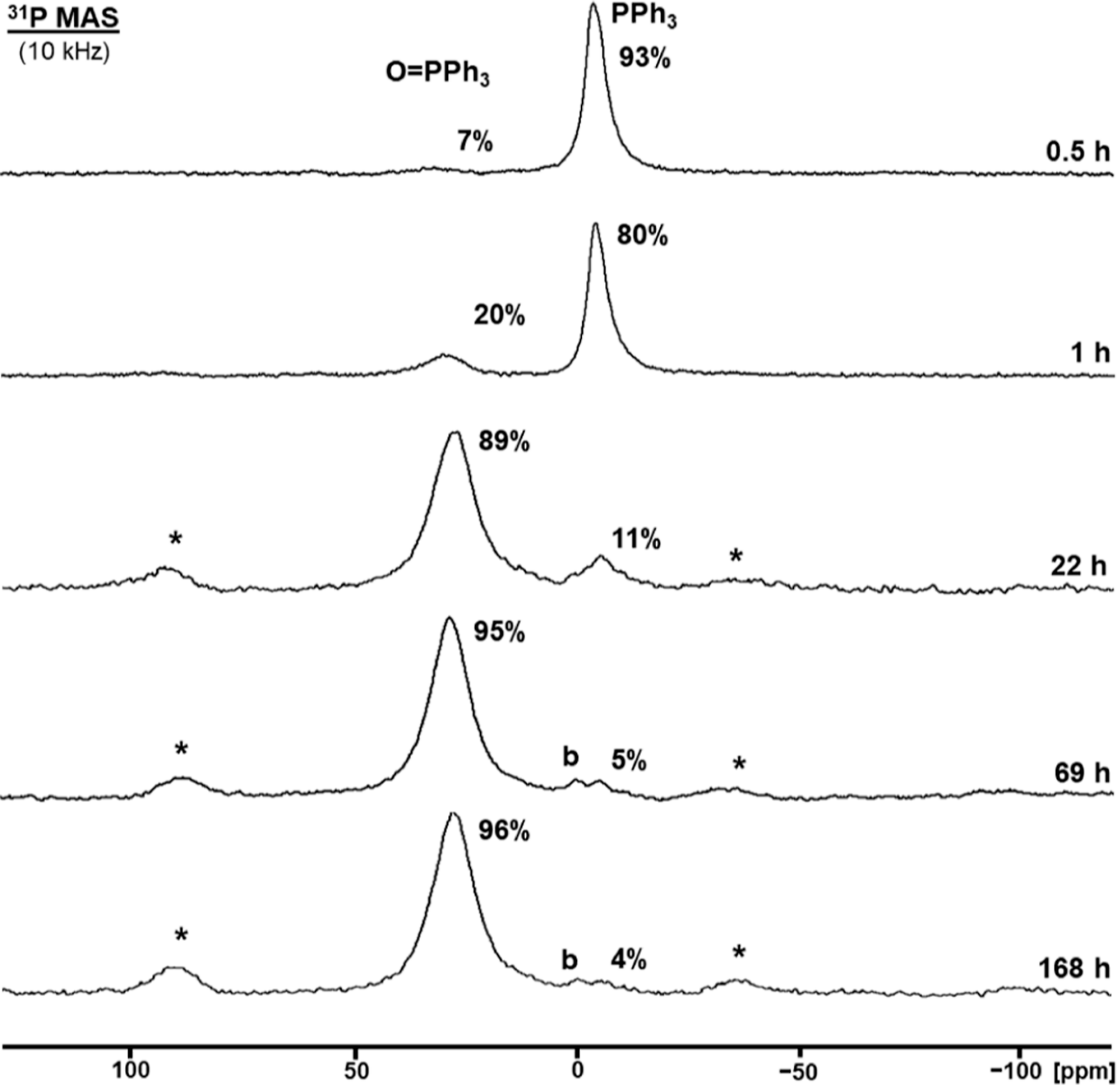
^31^P MAS NMR spectra of adsorbed PPh_3_ (**6**) on AC (98% surface coverage) measured after the indicated times. The asterisks denote rotational sidebands of the phosphine oxide signal. The label **b** indicates the AC phosphate background.

All adsorption procedures and solid-state NMR measurements of PPh_3_ outlined above have been performed under strict exclusion of oxygen. This is necessary because, much to our surprise, once adsorbed on AC, **6** is selectively oxidized in the atmosphere to O=PPh_3_ (**6**_**ox**_) within hours, as detected by ^31^P MAS NMR (Figure 4). The presence of P(V) species other than **6**_**ox**_ on the AC surface could be excluded by comparison with previous studies of adsorbed phosphine oxides,^48^ polycrystalline and adsorbed phosphonium salts,^79,80^ and phosphinic and phosphonic acids.^79^ The oxidation progress of adsorbed **6** has also been monitored using ^31^P MAS NMR (Figure 4). After only 1 h, 20% of adsorbed **6**_**ox**_ is already formed. The oxidation progresses quickly to reach 89% conversion after 1 day of exposure to the atmosphere. The rate then slows, and 96% conversion is achieved after 7 days. It will be demonstrated below, however, that a lower surface coverage of **6** on AC (40%) leads to much faster and complete oxidation to **6**_**ox**_ within about 3 h. The competition of **6** with the more strongly adsorbed **6**_**ox**_ on the surface^48^ could explain why 100% conversion is not achieved with a full monolayer surface coverage.

Importantly, no air oxidation has ever been observed for polycrystalline **6** and for PPh_3_ adsorbed on silica, and the samples remained unchanged over years when stored under the laboratory atmosphere.^10^ In order to generate the phosphine oxide O=PPh_3_ on silica, hydrogen peroxide or other strong oxidizing agents^10,79^ have to be applied, while on AC, the oxidation of **6** to **6**_**ox**_ takes place in air. Even after prolonged exposure to the atmosphere, **6**_**ox**_ adsorbed on the AC surface remains unchanged and does not form other P(V) species as outlined above. As a preliminary consideration regarding the reaction mechanism of the oxidation, discussed in more detail below, this means that the many side products that come into existence during the oxidation of phosphines in solution^36,49^ are formed during the initial encounter of the phosphine with O_2_ and not during prolonged exposure.

### 2.2 General Adsorption and Oxidation Procedure

Intrigued by the observation that PPh_3_ could be oxidized selectively in air once it was adsorbed on AC, we sought to investigate whether more air-sensitive phosphines could also be cleanly oxidized in the atmosphere after adsorption. Consequently, P^*n*^Bu_3_ (**1**) was selected as the representative phosphine for detailed adsorption and oxidation experiments because it had been reported earlier that it is prone to produce side products when oxidized in air.^36^ Control experiments confirmed that **1** was 97% pure prior to the oxidation experiments (Figure S3). When neat **1** was exposed to air for 30 min, only 56% O=P^*n*^Bu_3_ (**1**_**ox**_) was formed besides phosphinic and phosphonic ester side products. Then, **1** was dissolved in THF and exposed to air for 30 min, which gave similar results with 56% formation of **1**_**ox**_, 36% side products, and 8% of remaining **1** (Figure S3). The signals could be assigned based on literature values.^36,81^ These experiments confirmed that **1**, whether neat or in solution, could not selectively be oxidized to **1**_**ox**_ using air.

Phosphine **1** is liquid at room temperature. Therefore, **1** was added to AC to self-adsorb overnight under an inert atmosphere of N_2_ in a 150% surface coverage. The calculation was based on the amount of **1** needed for a 100% surface coverage, the maximal monolayer loading on the AC surface, which was estimated as outlined in the Experimental Section. The adsorbed phosphine **1** was then exposed to air for 4 h. To determine the outcome of the reaction, the AC loaded with adsorbed **1** was placed under an inert atmosphere again, and dry, oxygen-free THF was added to form a suspension. A sample of this suspension was then measured with ^31^P NMR without prior filtration or other manipulation to avoid further potential changes in product ratios. The signals in the resulting spectrum were of good quality (Figure S4) and could be integrated to determine the product yields. Self-adsorption of **1** led to 94% conversion to **1**_**ox**_ with the remaining 6% consisting of residual **1** (Table 1, entry **A**).

**Table 1:** Methods and Results for Determining the Optimal Adsorption and Oxidation Procedure Based on **1**^a^

exp.	surface coverage (%)	adsorption method	oxidation method	yield **1**_**ox**_ (%)
**A**	150	neat/solid	dry/ads.	94
**B**	150	THF/solution	in THF	53
**C**	150	THF/solution	dry/ads.	86
**C**_**opt**_	100	THF/solution	dry/ads.	100
**D**	500	THF/solution	dry/ads.	98

a Experiment **C**_**opt**_ results in 100% yield of clean **1**_**ox**_ within 30 min (Figure 5). Spectra for experiments **A–C** and **D** are displayed in Figure S4. 100% surface coverage corresponds to a full monolayer.

While the self-adsorption and oxidation of **1** was successful, we also adsorbed phosphines by applying their solutions to AC and then removing the solvents in vacuo. While this necessitates an organic solvent, it broadens the scope of the method. Diverse solid or viscous phosphines can be adsorbed more quickly from solution than via self-adsorption without a solvent. To test the adsorption from solution, **1** was dissolved in THF and applied to AC with 150% surface coverage under a nitrogen atmosphere. The mixture was then stirred for 20 min to ensure that the dissolved phosphine entered all pores of the AC. Two oxidation conditions were then tested. In the first, the suspension was exposed to air for 1 h. In the second, the THF was removed in vacuo, and the dry AC with adsorbed **1** was exposed to air for 1 h. The suspension oxidation method led to a yield of 53% **1**_**ox**_, 2% side products, and 45% residual **1** (Table 1, entry **B**). The oxidation of surface-adsorbed **1** in the absence of any solvent gave 86% **1**_**ox**_ and 14% **1** (Table 1, entry **C**). The ^31^P NMR spectra are displayed in Figure S4.

Given the higher yield and greater purity of **1**_**ox**_, the dry oxidation method (Table 1, entries **A**, **C**, **C**_**opt**_, and **D**) is clearly the superior method compared to oxidation in the presence of the solvent. The faster oxidation rate for **1** adsorbed on the surface of AC after removal of the solvent is due to the fact that the phosphine is exposed on the surface, and oxygen has immediate access to all phosphine molecules. In case the solvent is present during the oxidation step, oxygen has to diffuse through the liquid medium to reach the phosphines. Furthermore, for the clean oxidation of phosphines that are not air sensitive, both oxygen and phosphine have to be adsorbed on the AC surface (see below). This is less likely when the phosphine and oxygen must compete with the solvent for access to the AC surface.

To further assess the role of adsorbed O_2_ in the reaction, phosphine **1** was adsorbed on AC with a lower surface coverage (100%) prior to performing the dry oxidation method. In this experiment, 100% spectroscopic yield was achieved in only 0.5 h (Table 1, entry **C**_**opt**_). The ^31^P NMR spectrum of the obtained clean **1**_**ox**_ is displayed in Figure 5. It can be concluded that the surface coverage affects the reaction rate, with a faster reaction at lower phosphine coverage. However, it is worth noting that within the range studied here, the surface coverage does not significantly affect the selectivity. When a 5-fold monolayer of **1** was adsorbed on AC and the dry material oxidized overnight, a 98% spectroscopic yield of clean **1**_**ox**_ was obtained (Figure S4 and Table 1, entry **D**).

**Figure 5:**
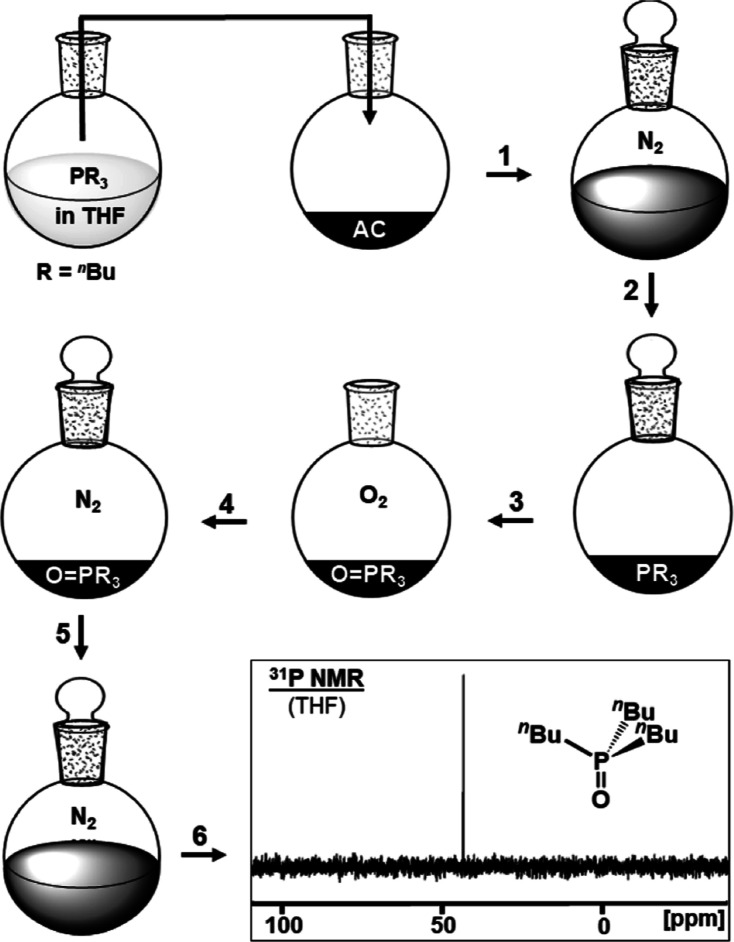
Optimized general adsorption and oxidation procedure depicted for P^*n*^Bu_3_ (**1**). Step 1: phosphine is adsorbed from solution under nitrogen. Step 2: solvent is removed in vacuo. Step 3: surface-adsorbed phosphine is oxidized in air. Step 4: AC with adsorbed phosphine oxide is placed under an inert atmosphere. Step 5: THF is used to create a suspension. Step 6: ^31^P NMR to determine the composition of the mixture. The spectrum corresponds to experiment **C**_**opt**_ in Table 1.

Given these results, all subsequent oxidation experiments were performed using adsorption of a submonolayer of phosphine from solution, followed by removal of the solvent in vacuo, and oxidation of the dry material by exposure to the atmosphere for 0.5 h (Figure 5).

It should be noted that at the beginning of these studies, we found that the ^31^P NMR spectrum of phosphines oxidized after adsorption on AC (Figure S5, top spectrum) showed a peak at about 3 ppm, which was assigned to residual phosphate in the AC based on the fact that phosphoric acid is used in the synthesis of AC.^60,61^ To remove the phosphate and exclude any role of it in the oxidation of the phosphines, the AC was washed vigorously with deionized H_2_O and dried in vacuo prior to use. To confirm the removal of the phosphate, a ^31^P NMR spectrum of the washings was recorded which showed the signal at about 3 ppm (Figure S5, bottom spectrum). In subsequent ^31^P NMR spectra of adsorbed phosphines and their oxides, only trace amounts of residual phosphate were detected. All oxidation reactions described in this work, except the one performed for Figure S5, were carried out with thoroughly washed AC. Any influence of the phosphate or phosphoric acid on the oxidations can therefore be excluded.

### 2.3 Reuse of AC and Phosphine Oxide Recovery

With the activity of the AC surface for phosphine oxidation confirmed and an optimal procedure determined, we next aimed to assess the reusability of the AC. While AC is not an expensive material, it is interesting with respect to mechanistic considerations whether the AC surface becomes less active in facilitating the oxidation of phosphines after one application. For this purpose, the AC material that had been used for the experiment **C**_**opt**_ (Table 1) was washed thoroughly with THF to remove adsorbed **1**_**ox**_. After one washing, the amount of **1**_**ox**_ was significantly reduced, and it was practically gone after the third washing, as demonstrated by ^31^P NMR of the AC suspension in THF (Figure S6). Adsorption and oxidation of **1** was then repeated six more times using this same sample of AC, and similar amounts of adsorbed **1**. The yields are determined by integrating the signals of **1** and **1**_**ox**_ as described in the previous section and reported in Table 2. Selected spectra are displayed in Figure S7.

**Table 2:** Spectroscopic Yields When the Same Sample of AC Was Used Repeatedly to Oxidize Batches of **1** to **1**_**ox**_^a^

cycle	surface coverage of **1** (%)	yield of **1**_**ox**_ (%)
**1**	100	100
**2**	105	100
**3**	96	73
**4**	84	65
**5**	91	48
**6**	77	52
**7**	86	33

a 100% surface coverage corresponds to a full monolayer.

Over seven adsorption and oxidation cycles using the same batch of AC, the yield of **1**_**ox**_ decreases (Table 2). The observed drop in activity could be due to traces of water in THF or air which gradually block the oxygen-activating sites on the AC surface. Alternatively, the feature of the AC supporting the oxidation might break down due to stirring, thus lowering the surface area. Despite the diminishing yield, the results show that the AC is, in principle, reusable over multiple cycles, should this be desirable. Since AC is an inexpensive material, the recycling experiments are, however, most interesting with respect to the mechanism of the selective oxidation.

As described in more detail in the Mechanistic Considerations section, the surface of AC consists primarily of aromatic ring systems that contain delocalized unpaired electrons.^69,70^ To assess whether the oxidation of phosphines on the AC surface uses up the unpaired electrons, EPR spectroscopy was applied. The EPR spectra of washed and dried pristine AC and the same AC batch after oxidation cycle 7 (Table 2) are practically identical (Figure S8). This confirms that the delocalized electrons are still present on the AC surface after the oxidation reactions. Furthermore, the fact that the EPR signal is a singlet indicates that the unpaired electrons reside delocalized in the aromatic system on the AC surface and do not stem from traces of a metal such as Fe.^69^ When diverse metals were adsorbed onto AC surfaces previously and investigated via EPR, more complex EPR splitting patterns of the signals as compared to unmodified AC were observed.^82−84^

Furthermore, we employed ^13^C CP/MAS (cross-polarization in combination with MAS)^11^ measurements to identify differences in the AC before phosphine adsorption and after seven phosphine adsorption and oxidation cycles (Figure S9). The dominant feature in both ^13^C CP/MAS spectra are the overlapping aromatic peaks at about 130 ppm with the characteristic large CSA (chemical shift anisotropy)^11,85^ that manifests in first-order rotational sidebands even at 10 kHz spinning speed. Importantly, the spectrum of the used material remains unchanged compared to that of the pristine AC. This result indicates that the aromatic hydrocarbons of the AC surface stay intact.

For practical applications of the surface-assisted oxidation method in synthesis, the yield of phosphine oxide is crucial. Therefore, with the selective surface-assisted phosphine oxidation and reusability of the AC confirmed, the recovery of **1**_**ox**_ from the AC samples was studied next. At this point, it should be noted that once the phosphine oxide is generated, no further oxidation occurs in air, which facilitates the workup as no inert atmosphere is needed after the surface-assisted oxidation. In a representative experiment for retrieving the phosphine oxide, an AC batch containing **1**_**ox**_ was stirred in a solvent for 30 min, and the suspension was gravity-filtered through filter paper. The dried AC was then removed from the filter paper and transferred back into the original reaction flask. The washing step was then repeated two more times, and the solvent was removed in vacuo to yield **1**_**ox**_. Four different solvents were evaluated using this procedure (Table 3). The purity of the retrieved phosphine oxide was high in each case, with up to 98%, as demonstrated by ^31^P, ^13^C, and ^1^H NMR spectra of **1**_**ox**_ (Figures S10 and S11), which matched the data reported earlier.^36^ Regarding the efficiency of recovering **1**_**ox**_, ethanol afforded the highest unoptimized yield of 75% (Table 3).

**Table 3:** Recovery of Adsorbed **1**_**ox**_ from AC by Washing with Diverse Solvents (10 mL of Solvent per 400 mg AC)^a^

surface coverage of **1** (%)	solvent	recovered yield of **1**_**ox**_ (%)	purity (%)
120	toluene	43	98
110	methanol	52	97
160	THF	59	96
110	ethanol	75	96

a The purity was determined by integration of the ^31^P NMR signals of the recovered material. 100% surface coverage corresponds to a full monolayer on the AC surface prior to oxidation.

Additional washes and producing larger batches for Soxhlet extraction of **1**_**ox**_ could improve its recovered yield. This is indicated because after the first two washing cycles that produced 74.7 mg (43%) and 42.7 mg (25%), the third still led to an additional 13.7 mg (7%) yield of **1**_**ox**_. A slightly varied procedure that minimizes the loss of product along with the AC on the filter paper increased the recovered yield of OPPh_3_ (**6**_**ox**_) to 92%, see Experimental Section.

### 2.4 General Applicability to Diverse Phosphines

To assess the general applicability of the adsorption/oxidation method, five more, widely used phosphines with different Tolman angles,^74,75^ as given in the caption of Figure 2, have been tested, PEt_3_, (**2**), P^*n*^Oct_3_ (**3**), PMe^*t*^Bu_2_ (**4**), PCy_3_ (**5**), and PPh_3_ (**6**) (Figure 2). We selected phosphines with varying Tolman angles to investigate whether those with larger angles (**4**–**6**) would be more prone to O_2_ attack from the top, leading to increased side product formation. On the other hand, phosphines with smaller Tolman angles (**1**–**3**) should preferentially react with surface-bound O_2_, resulting in the selective oxidation. Phosphines **2** and **3** were also selected to determine whether the methylene chain length would play a role in the surface-assisted reaction. It is conceivable that the long chains of **3**, that are assumedly also adsorbed and mobile on the surface as in the cases of phosphine oxides adsorbed on silica,^46^ could prevent the interaction of the P atom with coadsorbed oxygen. Phosphine **4** provides more insight because the different substituents could lead to an even greater diversity of side products during oxidation of the neat or dissolved phosphine and surface-assisted selective oxidation could have increased merit. Phosphine **5** was selected to see the impact of cyclic versus straight-chain substituents and because it is one of the most popular phosphines with large steric demand in coordination chemistry. Finally, unlike phosphines **2**–**5**, **6** is less electron rich and was chosen because it is very resistant to oxidation in air, even in solution.^49^ Its *V*_min_ value (kcal/mol) that describes the electron donating capability of a phosphorus center is much less negative (−34.85) than, for example, the values of **1** (−43.71), **2** (−43.51), and **5** (−45.48).^49,86^ However, the solid-state NMR investigation described above proved that, adsorbed on the AC surface, **6** is oxidized readily and selectively. In general, with the representative **6**, we chose a less electron-rich phosphine to probe whether the AC surface could potentially also facilitate the selective oxidation of electron-poor, robust triarylphosphines.^49^

Given a positive outcome of the surface-assisted oxidation of diverse phosphines **1**–**6**, the hitherto indispensable peroxides as oxidizers and entailing purification steps for obtaining the corresponding clean phosphine oxides could probably be avoided for all tertiary phosphines.^36^

Before any AC adsorption and oxidation of the phosphines, control experiments were undertaken for **2**–**6**, analogous to those performed for **1**. The phosphines were measured by ^31^P NMR prior to oxidation and checked for traces of pre-existing oxides; then, they were oxidized as neat substances in air and dissolved in THF. Phosphines **2**–**5** gave a mixture of the corresponding oxides **2**_**ox**_**-5**_**ox**_ and various side products. Only phosphine **6** showed no oxide signals, as expected (Table 4).^49^ Unexpectedly, phosphine **5** gave a relatively high yield of **5**_**ox**_ when oxidized in THF (84%); however, large amounts of side products were formed (16%) (Table 4). In contrast, after oxidizing the adsorbed phosphine **5** in air, only 4% of side products were obtained (see below). The spectra connected with the control experiments for phosphines **2**–**6** are displayed in Figures S12–S16.

**Table 4:** Survey of the Air Oxidation of Phosphines **1**–**6** under Different Conditions and Composition of Samples Prior to Oxidation^a^

phosphine	oxidation method	yields (%)				
		phosphine oxide	side products			phosphine
			*a*	*b*	*c*	
P^*n*^Bu_3_ (**1**)	prior to oxid.	4	0	0	0	96
	neat	56	27	15	2	0
	dissolved	56	26	2	8	8
	adsorbed	100	0	0	0	0
PEt_3_ (**2**)	preoxid.	2	1	0	0	97
	neat	47	21	20	0	12
	dissolved	48	25	9	6	12
	adsorbed	92	8	0	0	0
P^*n*^Oct_3_ (**3**)	prior to oxid.	0	0	0	0	100
	neat	40	14	6	1	39
	dissolved	75	9	2	3	11
	adsorbed	97	3	0	0	0
P^*t*^Bu_2_Me (**4**)	prior to oxid.	0	0	0	0	100
	neat	38	39	8	15	0
	dissolved	27	61	5	7	0
	adsorbed	9	0	0	0	91
PCy_3_ (**5**)	prior to oxid.	4	0	0	0	96
	neat	62	0	0	0	38
	dissolved	90	5	3	2	0
	adsorbed	96	2	0	2	0
PPh_3_ (**6**)	prior to oxid.	0	0	0	0	100
	neat	0	0	0	0	100
	dissolved	0	0	0	0	100
	adsorbed	9	0	0	0	91

a All oxidation reactions were stopped after 0.5 h of exposure to air. All phosphines were completely oxidized when given longer reaction times. The phosphines were adsorbed on AC with 90–100% surface coverage. Side products **a**, **b**, and **c** correspond to the phosphinic acid ester (O=P(OR)R_2_), the phosphonic acid ester (O=P(OR)_2_R), and traces of other products, respectively. The ^31^P NMR spectra for **1** are displayed in Figures 5 and S3 and those for **2–6** in Figures S12–S16.

After the oxidations of phosphines **2**–**6** under conventional conditions, neat and in solution, they were subjected to the adsorption/oxidation method (Table 4). All phosphines were adsorbed on AC and exposed to the atmosphere for 30 min. The spectroscopic yields were calculated using the standard method of measuring the ^31^P NMR spectra of the AC suspensions and integrating the signals (Figure 5). The results for these experiments are summarized together with those of the control experiments in Table 4, and all ^31^P NMR spectra are displayed in Figures S12–S16. The results for phosphine **1**, discussed previously, are added in Table 4 to allow for a complete comparison of the results. It should be noted that all phosphines reach 100% conversion eventually over time. However, limiting the time frame to 30 min helped to compare their oxidation rates.

The AC adsorption/air oxidation method works well for alkylphosphines **1**–**3** which are oxidized selectively with high yields of **1**_**ox**_**–3**_**ox**_ within half an hour. Interestingly, despite being extremely susceptible to oxidation in air, only 9% of **4** is oxidized within 30 min when adsorbed on AC. The steric demand of the tertiary butyl substituents may slow the oxidation.^49−51^ This steric effect could especially come into play on a surface in which **4** is oxidized by surface-adsorbed O_2_. The rate of oxidation of **4** on AC over a longer time will be further investigated in the next section. Finally, it is noteworthy that 9% yield of **6**_**ox**_ is achieved after adsorption, as **6** is not oxidized in air or in solution. Both cases of **4** and **6** demonstrate that the AC surface propagates the air oxidation.

In order to probe whether the effect of the AC surface on the air oxidation of phosphines is universal, besides the AC brand DARCO, another type of AC (Norit) was tested. The selective oxidation of **1** took place with the AC brand Norit and 21% conversion was found after 30 min (Figure S17). The lower yield within the given time frame could be due to the different surface area, porosity, or availability of unpaired electrons. Nevertheless, the Norit experiment shows that the selective oxidation of surface-adsorbed phosphines is not limited to one specific brand of AC.^71,72^ It should be noted, however, that no oxidation of **6** occurs on oxide surfaces such as silica,^10^ so AC surfaces in general are special for the surface-assisted oxidation of phosphines.

### 2.5 Mechanistic Considerations

For preliminary mechanistic considerations of the selective surface-assisted oxidation of the phosphines, some results from the previous sections can be evaluated. The factors influencing the reaction are the steric demand of the substituents at phosphorus, the surface coverage on the AC, and the nature of its surface. The slow oxidation rates of **4** and **6** in particular offer an opportunity to understand more about how oxidation occurs.

**Figure 6:**
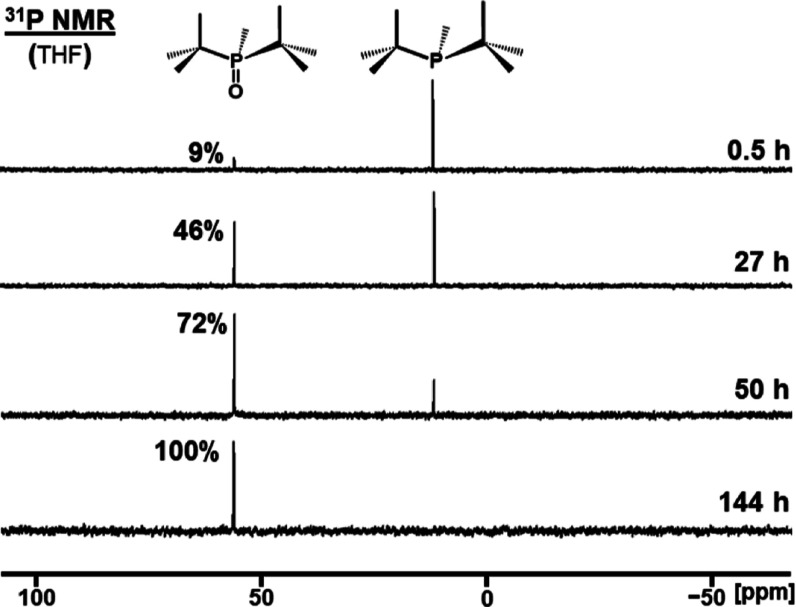
^31^P NMR spectra of **4** adsorbed on AC (93% surface coverage, standard procedure outlined in Figure 5) recorded after the indicated times of exposure to the atmosphere.

First, with phosphine **4**, the impact of the steric bulk around phosphorus can be investigated. Since only 9% of **4** was oxidized within 30 min, another experiment was performed in which the reaction was monitored over a longer time period. For this purpose, a small portion of the dried AC with adsorbed **4** was sampled, filled into an NMR tube, and placed under an inert atmosphere. Then, dry, oxygen-free THF was added directly to the NMR tube to produce a suspension for the measurement. This method allowed the mother batch of AC with absorbed **4** to be left undisturbed to further oxidize over days with small portions being removed for measurements at different time intervals. The spectra corresponding to these experiments are shown in Figure 6 together with the obtained spectroscopic yields.

As shown in Figure 6, the selective oxidation of **4** continues to progress past the initial measurement time at 30 min with 100% conversion achieved after about 6 days. This progress and the absence of side products show that the underlying mechanism responsible for the oxidation of all the adsorbed phosphines is still present for **4**, although the oxidation occurs at a slower rate. Since all phosphines were adsorbed with roughly the same surface coverage, this discrepancy must be due to the nature of **4**. From our control experiments, we also know that **4** oxidizes extremely readily in air, with no **4** remaining after 30 min of oxidation time, whether neat or dissolved in THF (Table 4). Therefore, the slower rate for adsorbed **4** must be due to the perpendicular orientation of the molecules of **4** with respect to the AC surface. Thus, we assume that the steric bulk of the tertiary butyl groups impedes the interaction between adsorbed O_2_ and **4**, slowing the rate of oxidation. Potential protection of the lone electron pair of **4** due to partial quaternization of the phosphine by interacting with the AC surface and therewith slower oxidation is unlikely because in this scenario, the trialkylphosphines **1**–**3** would display slow oxidation as well.

Similarly, the slow oxidation of **6** adsorbed on the AC surface makes it a useful probe for investigating the oxidation mechanism. As outlined for **4**, the oxidation of PPh_3_ adsorbed on AC with a 98% surface coverage was observed over time. The oxidation in this case was very slow (Figure S18 and Table S3) with a maximum yield of only 78% achieved after 214 h in air. Interestingly, the yield did not increase past this point and remained unchanged at the 263 h mark, when the final measurement was performed. Despite the slow rate, 78% yield is an important result because PPh_3_ cannot be oxidized in air without adsorption on AC.

One reason for the oxidation not progressing any further beyond a certain stage could be related to the high starting surface coverage of adsorbed **6**. The phosphine oxide, **6**_**ox**_, adsorbs strongly on the AC surface, as discussed above (Figure 4). Therefore, it is less mobile than adsorbed **6** and slows it down, decreasing its chance to interact with adsorbed O_2_. To confirm that **6**_**ox**_ interacts more strongly with the AC surface than **6**, a competition experiment was performed that tested the ability of **6**_**ox**_ to displace **6** from the surface. Phosphine **6** was adsorbed from solution as a full monolayer using the standard procedure (Figure 5). The dried material was then ground together with one equivalent of **6**_**ox**_ with a mortar and pestle for 5 min and measured by ^31^P MAS NMR (Figure S19). The signal of **6**_**ox**_ clearly stems from adsorbed molecules, as polycrystalline Ph_3_PO displays a larger CSA and small residual line width.^47^ Compared to a sample from the same adsorption batch without the added phosphine oxide, the signal of **6** broadens from 600 to 1100 Hz. It also moves upfield from −3.2 to −4.6 ppm, while the resonance of **6**_**ox**_ retains its chemical shift. The broadening suggests that the PPh_3_ signal is now composed of two signals which stem from adsorbed and polycrystalline **6**. Additionally, the upfield shift of the signal of **6** is consistent with going from an adsorbed to a nonadsorbed phosphine on the AC. Most importantly, the fact that **6**_**ox**_ is able to adsorb on AC that already hosts a full monolayer of **6** means that oxide **6**_**ox**_ binds more strongly to AC than **6** and has the potential to impede the surface adsorption of **6** and its interactions with surface-bound O_2_.

**Figure 7:**
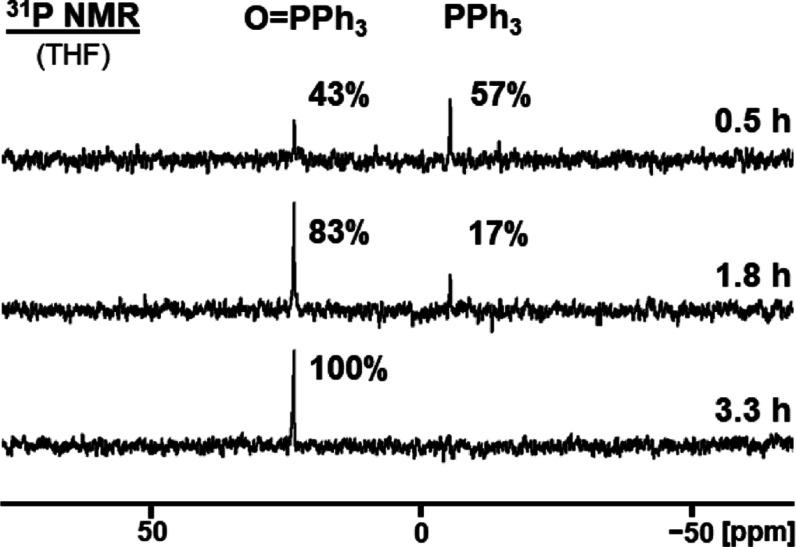
^31^P NMR spectra of adsorbed PPh_3_ (**6**) on AC (40% surface coverage) measured after the indicated times of exposure to the atmosphere.

To test the inverse correlation between the surface coverage and reaction rate observed previously for **1** with a phosphine that is oxidized more slowly, **6** was adsorbed on AC with lower surface coverages. Samples of **6** adsorbed on AC with 50% and 40% surface coverage were exposed to the atmosphere for 30 min. Compared to the 9% yield for 98% surface coverage, 50% coverage gave 26% yield, while 40% gave 43% yield after 30 min (Figures 7 and S20). The lower the surface coverage, the faster the oxidation progresses. This result indicates that the space available for O_2_ to adsorb on the AC surface and the mobility of the adsorbed phosphine molecules are important factors influencing the reaction rate.

The dry sample with 50% surface coverage of **6** was remeasured after being exposed to air for 24 h and showed 100% conversion to **6**_**ox**_ (Figure S20). Given the much faster oxidation rate for this sample, the 40% monolayer sample was also monitored over time with shorter time intervals between the measurements. The 40% surface coverage sample showed 83% conversion to **6**_**ox**_ after 1.8 h and 100% conversion after only 3.3 h (Figure 7). With a surface coverage of 40%, an isolated yield of 92% of **6**_**ox**_ could be realized by a modified workup procedure, as described in the Experimental Section (Figure S21).

All oxidation experiments described so far have been performed using the humid East Texas air. Therefore, water could have played a role in the oxidation step, either directly, or by stabilizing the generated phosphine oxides by forming hydrogen-bonded water adducts.^35−37^ However, when **1** is adsorbed on AC under strictly anhydrous conditions and oxidized with rigorously dried air, **1**_**ox**_ is generated selectively (Figure S22). Therefore, it can be concluded that water or moisture do not play a role in the surface-assisted oxidation of phosphines adsorbed on AC.

**Figure 8:**
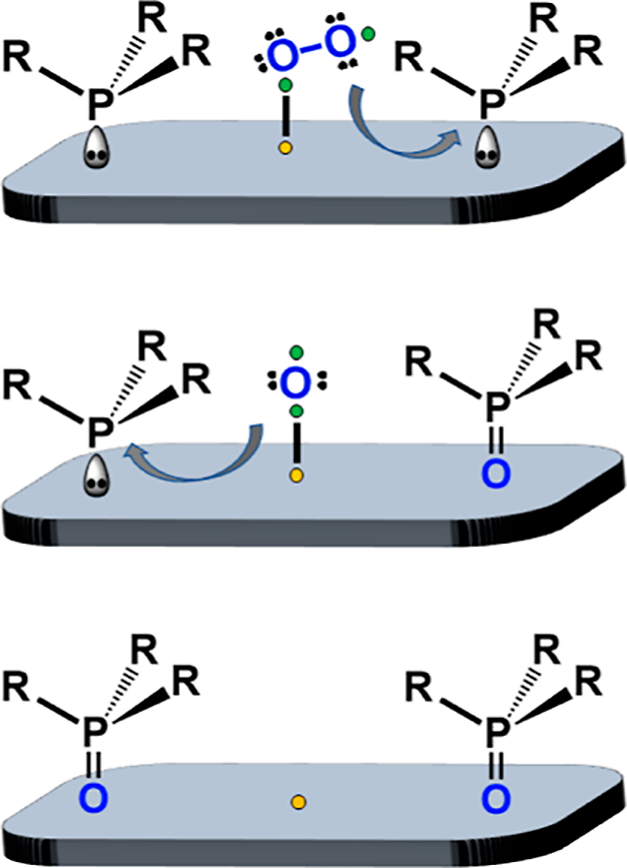
Proposed mechanism for the selective air oxidation of adsorbed phosphines on the surface of activated carbon (AC).

Given the results of the previously described experiments, a possible mechanism can be proposed (Figure 8). The ^31^P MAS experiments of adsorbed **6** prove that the phosphines are adsorbed on the AC surface and that the molecules are mobile (Figure 4). Residual polycrystalline PPh_3_ on the surface is not oxidized. Therefore, it can be concluded that adsorption is an indispensable prerequisite for the oxidation to occur. Thus, the phosphines are adsorbed to the AC during the oxidation reaction (Figure 8). The broad residual lines and reduced CSA of the signals of **6**_**ox**_ (Figure 4) furthermore prove that the phosphine oxides remain adsorbed on the AC surface (Figure 8).

Second, by varying the surface coverage of **6** on AC and monitoring the oxidation progress over time, we know that there is an inverse correlation between the phosphine surface coverage and the reaction rate (Figures 4 and 7). This suggests that the oxygen source for the oxidation reaction is also adsorbed on the AC surface. If the O_2_ came from the gas phase and reacted with the phosphines directly, we would not anticipate that the surface coverage significantly alters the reaction rate (Figures 4 and 7), as long as it does not exceed a monolayer. EPR spectroscopy proves that O_2_ readily interacts with the AC surface. When washed and dried AC is measured under a dried nitrogen atmosphere, its EPR signal is much broader and appears at a different field strength than the signal of AC when it is measured in air (Figure S23).

Since AC is known to contain unpaired electrons (Figure S8), we propose that the O_2_ adsorbs onto the AC via an interaction between the unpaired electron on the AC surface and one of the unpaired electrons of O_2_ as has been suggested before.^69−72^ Subsequently, the activated radical O_2_^•^ reacts with the lone electron pair of one phosphine to form the first phosphine oxide molecule, which remains adsorbed on the surface. This leaves an activated O^•^ atom behind which then reacts with the second phosphine (Figure 8). The high translational mobility of adsorbed phosphines on the AC surface, as proven above with solid-state NMR spectroscopy (Figures 3 and 4), facilitates the “sharing” of the O_2_ molecule between two phosphines. With the availability of the second phosphine, the insertion of the remaining O^•^ atom into a P–C bond of the phosphine oxide is less likely, and thus, the formation of side products is limited (Figure 1).

This proposed mechanism (Figure 8) also explains why the AC batch is reusable because the key radical is retained after multiple reaction cycles, as also proven by EPR spectroscopy (Figure S8). Furthermore, it explains why the formation of undesired side products with R–O–P bonds (Figure 1) is suppressed because the O_2_ is already interacting with the AC surface, limiting the formation of RO_2_^•^ radicals leading to these side products.^52^ Finally, this mechanism also provides an explanation for the slower oxidation of **4** on AC relative to straight-chain alkyl phosphines. The steric hindrance of the *tert*-butyl groups of **4** inhibit the interaction between adsorbed O_2_ and phosphine **4** on the surface that is necessary for the oxidation.

Furthermore, the clean oxidation of trialkylphosphines and the oxidation of air-stable PPh_3_ on the AC surface show that the latter is playing an active role in driving the oxidation. Solid-state NMR of adsorbed PPh_3_ confirms that the adsorption of the phosphine, as opposed to residing in the polycrystalline state in the AC pores, is necessary for the oxidation. An inverse correlation between the oxidation rate and surface coverage of the phosphine suggests that O_2_ adsorption is also an important prerequisite for the oxidation to proceed. In summary, we propose that O_2_ adsorbs from the air and is activated by unpaired electrons on the AC surface. The activated adsorbed O_2_ can then oxidize adsorbed phosphines to the phosphine oxide (Figure 8). Since the phosphine oxides are not taking up any more oxygen to form side products, it can be concluded that the latter come into existence during the initial encounter of neat or dissolved phosphines with O_2_. The AC surface eliminates this reaction pathway.

## 3 Conclusions

In this contribution, phosphines P^*n*^Bu_3_ (**1**), PEt_3_ (**2**), P^*n*^Oct_3_ (**3**), PMe^*t*^Bu_2_ (**4**), PCy_3_ (**5**), and PPh_3_ (**6**) were used to demonstrate the ability of the AC surface to assist in their clean oxidation to the corresponding phosphine oxides **1**_**ox**_–**6**_**ox**_. All oxidations could be accomplished at room temperature and with air as the oxidant. Solid-state NMR experiments proved that all phosphines were adsorbed on the AC surface and that their oxidation rate increased with decreasing surface coverage. P^*n*^Bu_3_ (**1**) was chosen as a challenging representative phosphine for optimizing the reaction conditions. It has been oxidized selectively to O=P^*n*^Bu_3_ (**1**_**ox**_) with quantitative conversion. After demonstrating that **1**_**ox**_ is easily recoverable in good yield and that the AC surface is reusable, the method was extended to other phosphines. Straight-chain alkylphosphines could be oxidized cleanly and rapidly in air when spread out on the AC surface, while sterically hindered phosphines and the air-stable phosphine **6** were oxidized more slowly. However, by applying a lower surface coverage of the phosphine adsorbed on the AC surface prior to the air oxidation step, even **6**_**ox**_ could be recovered as clean phosphine oxide in nearly quantitative yield. The phosphines that needed more time for the air oxidation process were used to elucidate the reaction mechanism. Based on the results, it was suggested that the adsorption of both O_2_ and the phosphine on the AC surface were required for the selective oxidation. Unpaired electrons on the AC surface play a key role in the oxidation mechanism. EPR spectroscopy showed that the unpaired electrons present on the surface stem from the aromatic network on the AC surface and not from traces of any metal. After using the same AC sample over multiple oxidation cycles, the radical remains unchanged, proving its role in the phosphine oxidation on the AC surface.

The presented research shows that clean phosphine oxides can be obtained by surface-assisted oxidation in the atmosphere, without the need for expensive and potentially hazardous oxidizing agents like H_2_O_2_^36^ or di(hydroperoxy)alkanes.^39−45^ The oxidation is therefore more benign, and the workup is much simpler than described previously^36^ because no H_2_O_2_ adducts of the phosphine oxides need to be destroyed.^36^ In future projects, we will explore the potential of the surface-assisted oxidation with AC for other relevant oxidation reactions such as aldehyde oxidation^45^ and epoxidations.^87^

In summary, the results described above deepen our understanding of adsorption and AC surface-assisted oxidation while also offering a convenient method for the fast and straightforward, safe, and inexpensive synthesis of research-relevant clean phosphine oxides.

## 4 Experimental Section

### 4.1 General Aspects and Activated Carbon

The activated carbon (AC) brand DARCO produced by Fisher Scientific (650 m^2^/g) was applied for the studies. Only one specifically mentioned experiment utilized Norit AC (RBAA-3, specific surface area 877 m^2^/g^88^). Prior to use, the AC was washed with water to remove traces of phosphoric acid so that its signal did not interfere with phosphine or phosphine oxide peaks in the ^31^P NMR spectra. For this purpose, the AC was added to a round-bottom flask and fully immersed in deionized water. The flask with this mixture was then heated to 60 °C and stirred for 3 h. After cooling to room temperature, the suspension was gravity-filtered through filter paper. The AC was then placed in a Schlenk flask, dried in vacuo at 100 °C for about 3 h, and stored under purified N_2_.^89^ All phosphines were used as received from the vendor without further purification. Tetrahydrofuran (THF)^90^ was obtained from a solvent distill and dried with molecular sieves prior to use. The Schlenk flasks were dried in an oven (130 °C) before use and kept under a N_2_ atmosphere using a Schlenk line.^89^ All reactions, as well as transfer and addition of materials, were performed under a purified N_2_ atmosphere. The air that was admitted eventually was not subjected to any special treatment.

### 4.2 Solution NMR

The NMR samples were prepared under a purified N_2_ atmosphere with dried, oxygen-free THF.^90^ For ^31^P NMR, nondeuterated THF^90^ was used while disabling the lock function. The ^13^C and ^31^P NMR measurements were performed with routine pulse programs and proton decoupling. The samples were measured on a Varian 500 MHz NMR with the external standard ClPPh_2_ (neat liquid, δ(^31^P) = +81.92 ppm) for ^31^P and the solvent signals for ^13^C. The ^31^P NMR spectra were typically obtained with satisfactory S/N ratio with 128 or 256 scans. Line broadening factors of up to 10 Hz were applied as needed.

### 4.3 Solid-State NMR

Solid-state NMR samples were prepared by packing the material into 4 mm rotors under air. All measurements were performed with a Bruker Avance 400 solid-state NMR instrument. The ^31^P NMR spectra were recorded using a standard single-pulse program with simple high-power decoupling and a 2 s pulse repetition time. 128 or 256 scans in combination with a line-broadening factor of 50 Hz typically resulted in sufficient spectrum quality. The T_1_ measurements were performed using a Bruker standard inversion recovery pulse program in combination with a rotational speed of 10 kHz to maximize the signal-to-noise ratio. The ^31^P solid-state NMR spectra were calibrated with the external standard (NH_4_)_2_HPO_4_ (δ(^31^P) = +0.81 ppm). ^13^C CP/MAS measurements of AC were performed using a standard Bruker RAMP sequence, and the spectra were calibrated with glycine as an external standard (δ(CO) = +176.5 ppm).

### 4.4 Oxidation Control Experiments

All phosphines, except PPh_3_, were stored under an inert atmosphere in an argon-operated glovebox. Prior to the adsorption experiments, three control experiments were performed for each phosphine. In the first, a portion of the phosphine was added to an NMR tube under an inert atmosphere and dissolved in dry, oxygen-free THF.^90^ In the second, a portion of the neat phosphine was exposed to the atmosphere in a fume hood for 30 min. The solid phosphines PCy_3_ and PPh_3_ were ground to a fine powder with a mortar and pestle to facilitate the contact with air. A portion of the exposed sample was then filled into an NMR tube, placed under an inert atmosphere, and then dissolved in dry, oxygen-free THF^90^ prior to the NMR measurement. In the third control experiment, ca. 2 mL of dry, oxygen-free THF^90^ was filled into a scintillation vial in air. A portion of the phosphine was then added directly to the THF,^90^ and the vial was left in a fume hood under air for 30 min. In the case of a solid phosphine, the solution was gently stirred until it was fully dissolved. A portion of the solution was then added to an NMR tube under an inert atmosphere to prevent further oxidation. All control samples were then measured with ^31^P solution NMR.

### 4.5 Estimating the Surface Coverages of Phosphines

To estimate phosphine surface coverages, an energy-minimized model of each phosphine (**1**–**6**) was created using Avogadro software. Hereby, the molecular radius was measured with the P atom as the central point. This radius, in combination with the reported surface area of the AC brand (DARCO), allowed the calculation of a maximum monolayer loading of the surface for each phosphine (Table S4). A full monolayer is referred to as a 100% surface coverage in this contribution.

### 4.6 Dry Adsorption

AC (216.9 mg) was placed under an inert atmosphere in an oven-dried Schlenk flask. Phosphine **1** (66.9 mg, 0.331 mmol, 153% surface coverage) was added to the AC under N_2_. The solid mixture was then stirred for 21 h under N_2_.

### 4.7 Adsorption from Solution

In a representative experiment, activated carbon (138.1 mg) was placed in a 100 mL Schlenk flask. Tri-*n*-butylphosphine (**1**, 30.6 mg, 0.151 mmol, 86% surface coverage) was filled into a separate 100 mL Schlenk flask^89^ and dissolved in THF^90^ (10 mL). The phosphine solution was then added to the AC, and the mixture was stirred for 20 min. The THF^90^ was removed in vacuo at 40 °C until the activated carbon was dry (∼30 min).^89^ The phosphine remained adsorbed on the AC surface. All samples were carefully dried in vacuo prior to the oxidation, except for the wet oxidation experiment (Table 1, Exp B), when the sample was exposed to air without removing THF.^90^

### 4.8 Solution NMR Measurements in the Presence of AC

The following representative procedure was applied for experiments in which the progress of the oxidation was checked only once. After oxidizing the AC-adsorbed phosphine in a Schlenk flask exposed to air, the sample was placed under a nitrogen atmosphere.^89^ Dry, oxygen-free THF^90^ (8 mL) was added, and the mixture was stirred for 5 min to wash the phosphine oxide and potentially remaining phosphine off of the AC surface. Subsequently, without filtration, a portion of the suspension of AC in the solution of the phosphine oxide in THF^90^ was added to an NMR tube under N_2_, and a ^31^P NMR spectrum was recorded. Spectroscopic yields were determined by integration of the signals using TopSpin software.

### 4.9 Monitoring the Oxidation of Adsorbed Phosphines

For experiments in which the progress of the oxidation of adsorbed phosphine was checked multiple times, the AC sample loaded with the phosphine was allowed to continue the oxidation in air. When the reaction was checked for oxidation progress by solution NMR, a portion of the dry AC was filled into an NMR tube and placed under an inert atmosphere. Dry, oxygen-free THF^90^ was then added, and the sample was measured immediately. When the reaction was checked for oxidation progress using solid-state NMR, a portion of the adsorbed AC material was packed into a solid-state NMR rotor and measured. After the first measurement, the rotor was opened, and its contents were exposed to the atmosphere in the fume hood until the rotor was recapped for the next monitoring measurement.

### 4.10 Maximizing the Yield of Phosphine Oxide **6**_**ox**_

PPh_3_ (**6**) (311.0 mg, 1.19 mmol) was adsorbed onto AC (1.7386 g, 40% surface coverage) following the standard method for adsorption from the solution described above. Adsorbed **6** was allowed to oxidize in air overnight to ensure complete conversion to O=PPh_3_ (**6**_**ox**_). Then, THF^90^ (30 mL) was added to the solid, and the mixture was stirred for 30 min at 40 °C. Subsequently, the suspension was gravity-filtered through filter paper, and the liquid was collected in a 300 mL round-bottom flask. The dried AC and the filter paper, cut into small pieces, were then placed into the original flask. This washing and filtering sequence was repeated two more times using fresh filter paper for each filtration and adding the used filter back to the original flask. THF^90^ was then removed in vacuo from the collected extracts to yield clean **6**_**ox**_ as a white powder (207.1 mg, 63% yield). The combined AC and filter paper mixture was again stirred overnight in ethanol (50 mL) at RT. The suspension was filtered, and ethanol removed in vacuo to yield additional **6**_**ox**_ (75.6 mg, 86% total recovery). The overnight wash and recovery were repeated one more time to recover an additional 21.8 mg of O=PPh_3_ (304.5 mg, 1.16 mmol, 92% total yield, Figure S21).

### 4.11 Washing and Recovery of the Activated Carbon

In a representative procedure for recovering the phosphine oxide from the AC surface, the washing solvent (10 mL) was added to the dry AC sample in a Schlenk flask, and the mixture was stirred for 30 min. The suspension was then transferred to a funnel with filter paper and allowed to gravity filter with the supernatant collected in a Schlenk flask. Once dry, the AC was transferred back into the original Schlenk flask. The washing and filtration steps were then repeated two more times for three total washing rounds. After completion of the washing cycles, the solvent was removed in vacuo at about 40–70 °C^89^ to yield the phosphine oxide.

### 4.12 Oxidation of Adsorbed Phosphine with Dry Air

AC (401.4 mg) was placed into an oven-dried Schlenk flask, dried in vacuo for 30 min at 90 °C, and placed under a dry nitrogen atmosphere.^89^ In a second oven-dried Schlenk flask, P^*n*^Bu_3_ (**1**) (29.4 mg, 0.145 mmol, 28% surface coverage) was dissolved in dry and oxygen-free THF^90^ (10 mL) under a dry nitrogen atmosphere. The phosphine solution was then transferred to the AC and stirred for 20 min under a dry nitrogen atmosphere. Subsequently, THF^90^ was removed in vacuo at 30 °C until the AC was dry, which took 25 min. Meanwhile, a plastic desiccator was filled with a mixture of drierite and molecular sieves (3 Å). Both desiccants had been dried in a drying oven at 250 °C for 3 h and dried at RT in vacuo for an additional h. For the oxidation of the adsorbed phosphine, a gentle stream of air, monitored by a bubbler, was blown through the desiccator and into the Schlenk flask containing **1** adsorbed on AC for 10 min. After the air flow was stopped, the flask was sealed to allow for an additional 50 min of oxidation time. Then, the AC with adsorbed **1**_**ox**_ was again placed under a dried nitrogen atmosphere, 4 mL of dry, degassed THF^90^ was added, and the mixture was stirred for 5 min. A portion of this suspension was then added to a dried NMR tube under a nitrogen atmosphere, and a ^31^P NMR spectrum was recorded (Figure S22).

## Supporting Information Available

Solution and solid-state NMR spectra; EPR spectra; data for T_1_ relaxation time determination; selected yields of oxidation experiments; and estimated monolayer surface coverages (PDF)

## References

[ref1] Pourhakkak P.; Taghizadeh A.; Taghizadeh M.; Ghaedi M.; Haghdoust S. Fundamentals of Adsorption Technology. In Adsorption: Fundamental Processes and Applications Ghaedi M., Ed.; Interface Science and Technology, 33; Academic Press: London, 2021; pp 1–70.

[ref2] Emoto S.; Isobe A.; Ikari T.; Kawamura K.; Kuroki S.; Naitoh M. Observation of Metal-free Phthalocyanine Adsorbed on SiC Reconstructed Surface. e-J. Surf. Sci. Nanotechnol. 2022, 20, 257–260. 10.1380/ejssnt.2022-040.

[ref3] Michalowska A.; Jedrzejewski K.; Kudelski A. Influence of the Co-Adsorbed Ions on the Surface-Enhanced Raman Scattering Spectra of Dopamine Adsorbed on Nanostructured Silver. Materials 2022, 15, 5972–5983. 10.3390/ma15175972. 36079352 PMC9457036

[ref4] Benzie J. W.; Harmon-Welch G. E.; Hoefler J. C.; Bakhmutov V. I.; Blümel J. Molecular Dynamics and Surface Interactions of Nickelocene Adsorbed on Silica: A Paramagnetic Solid-State NMR Study. Langmuir 2022, 38, 7422–7432. 10.1021/acs.langmuir.2c00301. 35675156

[ref5] Harmon-Welch G. E.; Hoefler J. C.; Trujillo M. R.; Bhuvanesh N.; Bakhmutov V. I.; Blümel J. Creating Solid Solutions of Metallocenes: Migration of Nickelocene into the Ferrocene Crystal Lattice in the Absence of a Solvent. J. Phys. Chem. C 2023, 127, 3059–3066. 10.1021/acs.jpcc.2c07441. PMC10848251 38333002

[ref6] Liu H.; Li H.; He Y.; Cheng P.; Zhang Y.; Feng B.; Li H.; Wu K.; Chen L. Condensation and asymmetric amplification of chirality in achiral molecules adsorbed on an achiral surface. Nat. Commun. 2023, 14, 2100 10.1038/s41467-023-37904-z. 37055409 PMC10101975

[ref7] Colabella J. M.; Stall R. A.; Sorenson C. T. The Adsorption and Subsequent Oxidation of AsH_3_ and PH_3_ on Activated Carbon. J. Cryst. Growth 1988, 92, 189–195. 10.1016/0022-0248(88)90449-6.

[ref8] Harris R. K.; Thompson T. V.; Norman P. R.; Pottage C. Adsorption competition onto activated carbon, studied by magic-angle spinning NMR. J. Chem. Soc., Faraday Trans. 1996, 92, 2615 10.1039/ft9969202615.

[ref9] Wyrick J.; Wang X.; Namboodiri P.; Kashid R. V.; Fei F.; Fox J.; Silver R. Enhanced Atomic Precision Fabrication by Adsorption of Phosphine into Engineered Dangling Bonds on H-Si Using STM and DFT. ACS Nano 2022, 16, 19114–19123. 10.1021/acsnano.2c08162. 36317737

[ref10] Hoefler J. C.; Yang Y.; Blümel J. Adsorption of solid phosphines on silica and implications for catalysts on oxide surfaces. New J. Chem. 2023, 47, 21190–21198. 10.1039/D3NJ03016D.

[ref11] Shenderovich I. G.; Limbach H.-H. Solid State NMR for Nonexperts: An Overview of Simple but General Practical Methods. Solids 2021, 2, 139–154. 10.3390/solids2020009.

[ref12] Schmidt-Rohr K.; Spiess H. W. Multidimensional Solid-State NMR and Polymers; Academic Press: London, UK, 1994.

[ref13] Samudrala K. K.; Huynh W.; Dorn R. W.; Rossini A. J.; Conley M. P. Formation of a Strong Heterogeneous Aluminum Lewis Acid on Silica. Angew. Chem., Int. Ed. 2022, 61, e202205745 10.1002/anie.202205745. 35951474

[ref14] Lamahewage S. N. S.; Atterberry B. A.; Dorn R. W.; Gi E.; Kimball M. R.; Blümel J.; Vela J.; Rossini A. J. Accelerated acquisition of wideline solid-state NMR spectra of spin 3/2 nuclei by frequency-stepped indirect detection experiments. Phys. Chem. Chem. Phys. 2024, 26, 5081–5096. 10.1039/D3CP05055F. 38259035

[ref15] Döller S. C.; Gutmann T.; Hoffmann M.; Buntkowsky G. A Case Study on the Influence of Hydrophilicity on the Signal Enhancement by Dynamic Nuclear Polarization. Solid-State NMR 2022, 122, 101829 10.1016/j.ssnmr.2022.101829. 36116176

[ref16] Zhang E.; Wu Y.; Shao H.; Klimavicius V.; Zhang H.; Taberna P. L.; Grothe J.; Buntkowsky G.; Xu F.; Simon P.; Kaskel S. Unraveling the Capacitive Charge Storage Mechanism of Nitrogen-Doped Porous Carbons by EQCM and ssNMR. J. Am. Chem. Soc. 2022, 144, 14217–14225. 10.1021/jacs.2c04841. 35914237

[ref17] Benzie J. W.; Bakhmutov V. I.; Blümel J. Benzene Adsorbed on Activated Carbon: A Comprehensive Solid-State Nuclear Magnetic Resonance Study of Interactions with the Pore Surface and Molecular Motions. J. Phys. Chem. C 2020, 124, 21532–21537. 10.1021/acs.jpcc.0c06225.

[ref18] Bonfante S.; Lorber C.; Lynam J. M.; Simonneau A.; Slattery J. M. Metallomimetic C-F activation catalysis by simple phosphines. J. Am. Chem. Soc. 2024, 146, 2005–2014. 10.1021/jacs.3c10614. 38207215 PMC10811696

[ref19] Etter M. C.; Baures P. W. Triphenylphosphine Oxide as a Crystallization Aid. J. Am. Chem. Soc. 1988, 110, 639–640. 10.1021/ja00210a076.

[ref20] Ashirov R.; Kimball M. R.; O’Brien M.; Bhuvanesh N.; Blümel J. Aluminum Trichloride Adducts of Phosphine Oxides: Structures, Solid-State NMR and Application. Inorg. Chim. Acta 2024, 564, 121952 10.1016/j.ica.2024.121952.

[ref21] Kharel S.; Jia T.; Bhuvanesh N.; Reibenspies J. H.; Blümel J.; Gladysz J. A. A Non-templated Route to Macrocyclic Dibridgehead Diphosphorus Compounds: Crystallographic Characterization of a ″Crossed-Chain″ Variant of *in*/*out* Stereoisomers. Chem.—Asian J. 2018, 13, 2632–2640. 10.1002/asia.201800739. 29870152

[ref22] Chrzanowski J.; Krasowska D.; Drabowicz J. Synthesis of Optically Active Tertiary Phosphine Oxides: A Historical Overview and the Latest Advances. Heteroat. Chem. 2018, 29, e21476 10.1002/hc.21476.

[ref23] Fletcher M. D. In Organophosphorus Reagents Murphy P. J., Ed.; Oxford University Press, 2004; pp 171–214.

[ref24] Adams H.; Collins R. C.; Jones S.; Warner C. J. A. Enantioselective Preparation of P-Chiral Phosphine Oxides. Org. Lett. 2011, 13, 6576–6579. 10.1021/ol202916j. 22106882

[ref25] Swamy K. C. K.; Kumar N. N. B.; Balaraman E.; Kumar K. V. Mitsunobu and Related Reactions: Advances and Applications. Chem. Rev. 2009, 109, 2551–2651. 10.1021/cr800278z. 19382806

[ref26] Dembinski R. Recent Advances in the Mitsunobu Reaction: Modified Reagents and the Quest for Chromatography-Free Separation. Eur. J. Org Chem. 2004, 2004, 2763–2772. 10.1002/ejoc.200400003.

[ref27] Beddoe R. H.; Andrews K. G.; Magne V.; Cuthbertson J. D.; Saska J.; Shannon-Little A. L.; Shanahan S. E.; Sneddon H. F.; Denton R. M. Redox-neutral organocatalytic Mitsunobu reactions. Science 2019, 365, 910–914. 10.1126/science.aax3353. 31467220

[ref28] Zheng A.; Liu S.-B.; Deng F. ^31^P NMR Chemical Shifts of Phosphorus Probes as Reliable and Practical Acidity Scales for Solid and Liquid Catalysts. Chem. Rev. 2017, 117, 12475–12531. 10.1021/acs.chemrev.7b00289. 28952317

[ref29] Zasukhin D. S.; Kasyanov I. A.; Kolyagin Y. G.; Bulygina A. I.; Kharas K. C.; Ivanova I. I. Evaluation of Zeolite Acidity by ^31^P MAS NMR Spectroscopy of Adsorbed Phosphine Oxides: Quantitative or Not?. ACS Omega 2022, 7, 12318–12328. 10.1021/acsomega.2c00804. 35449977 PMC9016808

[ref30] Wilmsmeyer A. R.; Gordon W. O.; Davis E. D.; Mantooth B. A.; Lalain T. A.; Morris J. R. Multifunctional Ultra-High Vacuum Apparatus for Studies of the Interactions of Chemical Warfare Agents on Complex Surfaces. Rev. Sci. Instrum. 2014, 85, 014101 10.1063/1.4846656. 24517783

[ref31] Bewick N. A.; Arendt A.; Li Y.; Szafert S.; Lis T.; Wheeler K. A.; Young J.; Dembinski R. Synthesis and Solid-State Structure of (4-Hydroxy-3,5-diiodophenyl)phosphine Oxides. Dimeric Motifs with the Assistance of O-H···O=P Hydrogen Bonds. Curr. Org. Chem. 2015, 19, 469–474. 10.2174/1385272819666141231000247.

[ref32] Burke N. J.; Burrows A. D.; Mahon M. F.; Warren J. E. Hydrogen Bond Network Structures Based on Sulfonated Phosphine Ligands: The Effects of Complex Geometry, Cation Substituents and Phosphine Oxidation on Guanidinium Sulfonate Sheet Formation. Inorg. Chim. Acta 2006, 359, 3497–3506. 10.1016/j.ica.2006.01.008.

[ref33] Joshi R.; Pasilis S. P. The Effect of Tributylphosphate and Tributylphosphine Oxide on Hydrogen Bonding Interactions between Water and the 1-Ethyl-3-methylimidazolium Cation in 1-Ethyl-3-Methylimidazolium bis(trifluoromethylsulfonyl)imide. J. Mol. Liq. 2015, 209, 381–386. 10.1016/j.molliq.2015.05.042.

[ref34] Tupikina E. Y.; Bodensteiner M.; Tolstoy P. M.; Denisov G. S.; Shenderovich I. G. P=O Moiety as an Ambidextrous Hydrogen Bond Acceptor. J. Phys. Chem. C 2018, 122, 1711–1720. 10.1021/acs.jpcc.7b11299.

[ref35] Begimova G.; Tupikina E. Y.; Yu V. K.; Denisov G. S.; Bodensteiner M.; Shenderovich I. G. Effect of Hydrogen Bonding to Water on the ^31^P Chemical Shift Tensor of Phenyl- and Trialkylphosphine Oxides and α-Amino Phosphonates. J. Phys. Chem. C 2016, 120, 8717–8729. 10.1021/acs.jpcc.6b01140.

[ref36] Hilliard C. R.; Bhuvanesh N.; Gladysz J. A.; Blümel J. Synthesis, purification, and characterization of phosphine oxides and their hydrogen peroxide adducts. Dalton Trans. 2012, 41, 1742–1754. 10.1039/C1DT11863C. 22159182

[ref37] Arp F. F.; Bhuvanesh N.; Blümel J. Hydrogen peroxide adducts of triarylphosphine oxides. Dalton Trans. 2019, 48, 14312–14325. 10.1039/C9DT03070K. 31475705

[ref38] Kharel S.; Bhuvanesh N.; Gladysz J. A.; Blümel J. New Hydrogen Bonding Motifs of Phosphine Oxides with a Silanediol, a Phenol, and Chloroform. Inorg. Chim. Acta 2019, 490, 215–219. 10.1016/j.ica.2019.03.019.

[ref39] Hoefler J. C.; Vu A.; Perez A. J.; Blümel J. Immobilized di(hydroperoxy)propane adducts of phosphine oxides as traceless and recyclable oxidizing agents. Appl. Surf. Sci. 2023, 629, 157333 10.1016/j.apsusc.2023.157333.

[ref40] Ahn S. H.; Cluff K. J.; Bhuvanesh N.; Blümel J. Hydrogen Peroxide and Di(hydroperoxy)propane Adducts of Phosphine Oxides as Stoichiometric and Soluble Oxidizing Agents. Angew. Chem. 2015, 127, 13539–13543. 10.1002/ange.201505291. 26457679

[ref41] Ahn S. H.; Bhuvanesh N.; Blümel J. Di(hydroperoxy)alkane Adducts of Phosphine Oxides: Safe, Solid, Stoichiometric, and Soluble Oxidizing Agents. Chem.—Eur. J. 2017, 23, 16998–17009. 10.1002/chem.201703676. 28853180

[ref42] Ahn S. H.; Lindhardt D.; Bhuvanesh N.; Blümel J. Di(hydroperoxy)cycloalkanes Stabilized via Hydrogen Bonding by Phosphine Oxides: Safe and Efficient Baeyer-Villiger Oxidants. ACS Sustainable Chem. Eng. 2018, 6, 6829–6840. 10.1021/acssuschemeng.8b00652.

[ref43] Arp F. F.; Ahn S. H.; Bhuvanesh N.; Blümel J. Selective synthesis and stabilization of peroxides *via* phosphine oxides. New J. Chem. 2019, 43, 17174–17181. 10.1039/C9NJ04858H.

[ref44] Arp F. F.; Bhuvanesh N.; Blümel J. Di(hydroperoxy)cycloalkane Adducts of Triarylphosphine Oxides: A Comprehensive Study Including Solid-State Structures and Association in Solution. Inorg. Chem. 2020, 59, 13719–13732. 10.1021/acs.inorgchem.0c02087. 32866378

[ref45] Arp F. F.; Ashirov R.; Bhuvanesh N.; Blümel J. Di(hydroperoxy)adamantane Adducts: Synthesis, Characterization and Application as Oxidizers for the Direct Esterification of Aldehydes. Dalton Trans. 2021, 50, 15296–15309. 10.1039/D1DT03243G. 34636381

[ref46] Kharel S.; Cluff K. J.; Bhuvanesh N.; Gladysz J. A.; Blümel J. Structures and Dynamics of Secondary and Tertiary Alkylphosphine Oxides Adsorbed on Silica. Chem.—Asian J. 2019, 14, 2704–2711. 10.1002/asia.201900632. 31168965

[ref47] Hilliard C. R.; Kharel S.; Cluff K. J.; Bhuvanesh N.; Gladysz J. A.; Blümel J. Structures and Unexpected Dynamic Properties of Phosphine Oxides Adsorbed on Silica Surfaces. Chem.—Eur. J. 2014, 20, 17292–17295. 10.1002/chem.201404880. 25394806

[ref48] Hubbard P. J.; Benzie J. W.; Bakhmutov V. I.; Blümel J. Disentangling different modes of mobility for triphenylphosphine oxide adsorbed on alumina. J. Chem. Phys. 2020, 152, 054718 10.1063/1.5142568. 32035468

[ref49] Barder T. E.; Buchwald S. L. Rationale Behind the Resistance of Dialkylbiaryl Phosphines toward Oxidation by Molecular Oxygen. J. Am. Chem. Soc. 2007, 129, 5096–5101. 10.1021/ja0683180. 17388595

[ref50] Zhang D.; Celaje J. A.; Agua A.; Doan C.; Stewart T.; Bau R.; Selke M. Photooxidation of Mixed Aryl and Biarylphosphines. Org. Lett. 2010, 12, 3100–3103. 10.1021/ol101122u. 20527907 PMC2892562

[ref51] Stewart B.; Harriman A.; Higham L. J. Predicting the Air Stability of Phosphines. Organometallics 2011, 30, 5338–5343. 10.1021/om200070a.

[ref52] Buckler S. A. Autoxidation of Trialkylphosphines. Org. Biol. Chem. 1962, 84, 3093–3097. 10.1021/ja00875a011.

[ref53] Kendall A. J.; Salazar C. A.; Martino P. F.; Tyler D. R. Direct Conversion of Phosphonates to Phosphine Oxides: An Improved Synthetic Route to Phosphines Including the First Synthesis of Methyl JohnPhos. Organometallics 2014, 33, 6171–6178. 10.1021/om500854u.

[ref54] Rinehart N. I.; Kendall A. J.; Tyler D. R. A Universally Applicable Methodology for the Gram-Scale Synthesis of Primary, Secondary, and Tertiary Phosphines. Organometallics 2018, 37, 182–190. 10.1021/acs.organomet.7b00684.

[ref55] Oishi T.; Lugo-Fuentes L. I.; Jing Y.; Jimenez-Halla J. O. C.; Barroso-Flores J.; Nakamoto M.; Yamamoto Y.; Tsunoji N.; Shang R. Proton to hydride umpolung at a phosphonium center *via* electron relay: a new strategy for main-group based water reduction. Chem. Sci. 2021, 12, 15603–15608. 10.1039/D1SC05135K. 35003590 PMC8654027

[ref56] Cluff K. J.; Blümel J. Adsorption of Ferrocene on Carbon Nanotubes, Graphene, and Activated Carbon. Organometallics 2016, 35, 3939–3948. 10.1021/acs.organomet.6b00691.

[ref57] Hubbard P. J.; Benzie J. W.; Bakhmutov V. I.; Blümel J. Ferrocene Adsorbed on Silica and Activated Carbon Surfaces: A Solid-State NMR Study of Molecular Dynamics and Surface Interactions. Organometallics 2020, 39, 1080–1091. 10.1021/acs.organomet.9b00800.

[ref58] Zhang X.; Gao B.; Creamer A. E.; Cao C.; Li Y. Adsorption of VOCs onto engineered carbon materials: A review. J. Hazard. Mater. 2017, 338, 102–123. 10.1016/j.jhazmat.2017.05.013. 28535479

[ref59] Pui W. K.; Yusoff R.; Aroua M. K. A review on activated carbon adsorption for volatile organic compounds (VOCs). Rev. Chem. Eng. 2019, 35, 649–668. 10.1515/revce-2017-0057.

[ref60] Cheng H. N.; Wartelle L. H.; Klasson K. T.; Edwards J. C. Solid-state NMR and ESR studies of activated carbons produced from pecan shells. Carbon 2010, 48, 2455–2469. 10.1016/j.carbon.2010.03.016.

[ref61] Puziy A. M.; Poddubnaya O. I.; Socha R. P.; Gurgul J.; Wisniewski M. XPS and NMR studies of phosphoric acid activated carbons. Carbon 2008, 46, 2113–2123. 10.1016/j.carbon.2008.09.010.

[ref62] Mikhalovsky S. V.; Zaitsev Y. P. Catalytic Properties of Activated Carbons I. Gas-Phase Oxidation of Hydrogen Sulphide. Carbon 1997, 35, 1367–1374. 10.1016/S0008-6223(97)00104-8.

[ref63] Aguilar C.; Garcia R.; Soto-Garrido G.; Arraigada R. Catalytic oxidation of aqueous methyl- and dimethylamines by activated carbon. Top. Catal. 2005, 33, 201–206. 10.1007/s11244-005-2528-y.

[ref64] Wang X.; Ning P.; Shi Y.; Jiang M. Adsorption of low concentration phosphine in yellow phosphorus off-gas by impregnated activated carbon. J. Hazard. Mater. 2009, 171, 588–593. 10.1016/j.jhazmat.2009.06.046. 19656624

[ref65] Sousa J. P. S.; Pereira M. F. R.; Figueiredo J. L. Modified activated carbon as catalyst for NO oxidation. Fuel Process. Technol. 2013, 106, 727–733. 10.1016/j.fuproc.2012.10.008.

[ref66] Fang Z.; Yu X.; Tu S. Catalytic oxidation of NO on activated carbons. Energy Procedia 2019, 158, 2366–2371. 10.1016/j.egypro.2019.01.285.

[ref67] Yu Q.; Li M.; Ning P.; Yi H.; Tang X. Characterization of Metal Oxide-modified Walnut-shell Activated Carbon and Its Application for Phosphine Adsorption: Equilibrium, Regeneration, and Mechanism Studies. J. Wuhan Univ. Technol., Mater. Sci. Ed. 2019, 34, 487–495. 10.1007/s11595-019-2078-y.

[ref68] Zhu J.; Li G.; Wang Q.; Zhou Y.; Wang J. Engineering Surface Groups of Commercially Activated Carbon for Benzene Hydroxylation to Phenol with Dioxygen. Ind. Eng. Chem. Res. 2019, 58, 20226–20235. 10.1021/acs.iecr.9b03889.

[ref69] Piette H. L. Chemical Applications of EPR. In NMR and EPR Spectroscopy. NMR-EPR Staff of Varian Associates; Pergamon Press: London, 1960; pp 207–223.

[ref70] Kimura M.; Miyamoto I. Discovery of the Activated-Carbon Radical AC^+^ and the Novel Oxidation-Reactions Comprising the AC/AC^+^ Cycle as a Catalyst in an Aqueous Solution. Bull. Chem. Soc. Jpn. 1994, 67, 2357–2360. 10.1246/bcsj.67.2357.

[ref71] Kawabata H.; Hayashi M. Benzylic oxygenation of alkylarenes with molecular oxygen in the presence of activated carbon. Tetrahedron Lett. 2004, 45, 5457–5459. 10.1016/j.tetlet.2004.05.030.

[ref72] Hayashi M. Oxidation Using Activated Carbon and Molecular Oxygen System. Chem. Rec. 2008, 8, 252–267. 10.1002/tcr.20152. 18752318

[ref73] Li Y.; Kolasinski K. W.; Zare R. N. Silica particles convert thiol-containing molecules to disulfides. Proc. Natl. Acad. Sci. U.S.A. 2023, 120, e2304735120 10.1073/pnas.2304735120. 37590411 PMC10450441

[ref74] Jover J.; Cirera J. Computational assessment on the Tolman cone angles for P-ligands. Dalton Trans. 2019, 48, 15036–15048. 10.1039/C9DT02876E. 31513203

[ref75] Min W. J.; Jung S.; Lim S. J.; Kim Y.; Shin S. K. Collision-Induced Dissociation of II-VI Semiconductor Nanocrystal Precursors, Cd^2+^ and Zn^2+^ Complexes with Trioctylphosphine Oxide, Sulfide, and Selenide. J. Phys. Chem. A 2009, 113, 9588–9594. 10.1021/jp905153v. 19658381

[ref76] Davies J. A.; Dutremez S. Solid state ^31^P NMR spectroscopic studies of tertiary phosphines and their complexes. Chem. Coord. Rev. 1992, 114, 61–98. 10.1016/0010-8545(92)80017-L.

[ref77] Harris R. K.; Merwin L. H.; Hagele G. Solid-state Nuclear Magnetic Resonance Study of a Series of Phosphonic and Phosphinic Acids. J. Chem. Soc. Faraday Trans. 1 1989, 85, 1409 10.1039/f19898501409.

[ref78] Sharma R.; Holland G. P.; Solomon V. C.; Zimmermann H.; Schiffenhaus S.; Amin S. A.; Buttry D. A.; Yarger J. L. NMR Characterization of Ligand Binding and Exchange Dynamics in Triphenylphosphine-Capped Gold Nanoparticles. J. Phys. Chem. C 2009, 113, 16387–16393. 10.1021/jp905141h.

[ref79] Blümel J. Reactions of Phosphines with Silicas: A Solid-State NMR Study. Inorg. Chem. 1994, 33, 5050–5056. 10.1021/ic00100a033.

[ref80] Sommer J.; Yang Y.; Rambow D.; Blümel J. Immobilization of Phosphines on Silica: Identification of Byproducts via ^31^P CP/MAS Studies of Model Alkyl-, Aryl-, and Ethoxyphosphonium Salts. Inorg. Chem. 2004, 43, 7561–7563. 10.1021/ic049065j. 15554612

[ref81] Stankevic M.; Pisklak J.; Wlodarczyk K. Aryl group – a leaving group in arylphosphine oxides. Tetrahedron 2016, 72, 810–824. 10.1016/j.tet.2015.12.043.

[ref82] Yao Y.; Wang L.; Sun L.; Zhu S.; Huang Z.; Mao Y.; Lu W.; Chen W. Efficient removal of dyes using heterogeneous Fenton catalysts based on activated carbon fibers with enhanced activity. Chem. Eng. Sci. 2013, 101, 424–431. 10.1016/j.ces.2013.06.009.

[ref83] Wang L.; Yao Y.; Sun L.; Mao Y.; Lu W.; Huang S.; Chen W. Rapid removal of dyes under visible irradiation over activated carbon fibers supported Fe(III)-citrate at neutral pH. Sep. Pur. Technol. 2014, 122, 449–455. 10.1016/j.seppur.2013.11.029.

[ref84] Meng X.; Ren Y.; Zhang X.; Liu J.; Ding Y.; Gao G.; Jiang W. Study on 3D electrochemical degradation of tetracycline hydrochloride by using Fe/Cu/Mn trimetal-doped granular activated carbon: Reactor, mechanism and regeneration. Sep. Pur. Technol. 2023, 324, 124526–124539. 10.1016/j.seppur.2023.124526.

[ref85] Duncan T. M. A Compilation of Chemical Shift Anisotropies; Farragut Press: Chicago, IL, 1990.

[ref86] Suresh C. H.; Koga N. Quantifying the Electronic Effect of Substituted Phosphine Ligands via Molecular Electrostatic Potential. Inorg. Chem. 2002, 41, 1573–1578. 10.1021/ic0109400. 11896726

[ref87] Chung M.; Maalouf J. H.; Adams J. S.; Jiang C.; Roman-Leshkov Y.; Manthiram K. Direct Propylene Epoxidation via Water Activation over Pd-Pt Electrocatalysts. Science 2024, 383, 49–55. 10.1126/science.adh4355. 38175873

[ref88] Chiang Y.; Chiang P.; Chang E. Evaluations of the physicochemical characterizations of activated carbons. J. Environ. Sci. Health 1998, 33, 1437–1463. 10.1080/10934529809376797.

[ref89] Caution! Extreme care should be taken both in the handling of the cryogen liquid nitrogen and its use in the Schlenk line trap to avoid the condensation of oxygen from air.

[ref90] Caution! Tetrahydrofurane (THF) is a highly flammable liquid that can easily be ignited by heat, sparks or flames.

