# Supplementary material for: Surface-Assisted Selective Air Oxidation of Phosphines Adsorbed on Activated Carbon
Source: Inorg Chem. 2024 May 9;63(20):9275–87. doi: 10.1021/acs.inorgchem.4c01027 (PMC11110008; doi:10.1021/acs.inorgchem.4c01027)
Supplement: Supplementary file 1 — ic4c01027_si_001.pdf [file ic4c01027_si_001.pdf]

Supplementary Information (SI)

Surface-Assisted Selective Air Oxidation of Phosphines Adsorbed on Activated Carbon

John C. Hoefler, Devin Jackson, Janet Blümel*

Department of Chemistry, Texas A&M University, College Station, TX, 77842-3012, USA.
Email: bluemel@tamu.edu

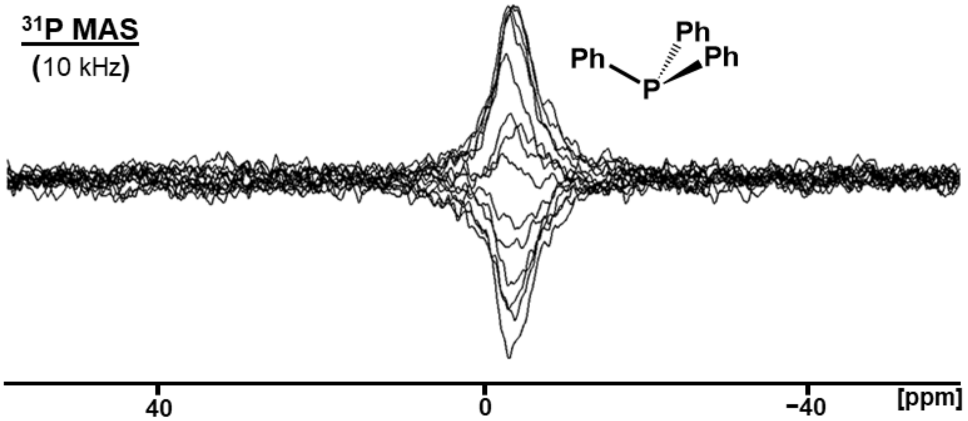

Figure S1. ^{31}P MAS NMR spectra acquired with a 180°-τ-90° inversion recovery pulse program for **6** adsorbed on AC with 98% surface coverage.

Table S1. Data for the inversion recovery NMR spectra in Figure S1, used for the fitting in Figure S2. The delay times τ between the 180° and 90° pulses are matched with the intensities of the resulting spectra which were measured using TopSpin software with arbitrary units. The data was normalized with every intensity value divided by the intensity value for a delay time of 0.0001 seconds and multiplied by -1.

**Delay Time τ (s)**	**Intensity**
0.0001	-1.000
0.001	-0.791
0.003	-0.725
0.03	-0.582
0.07	-0.373
0.1	-0.275
0.2	0.139
0.25	0.292
0.3	0.362
0.5	0.708
1	0.928
2	0.971
6	0.970
8	0.975

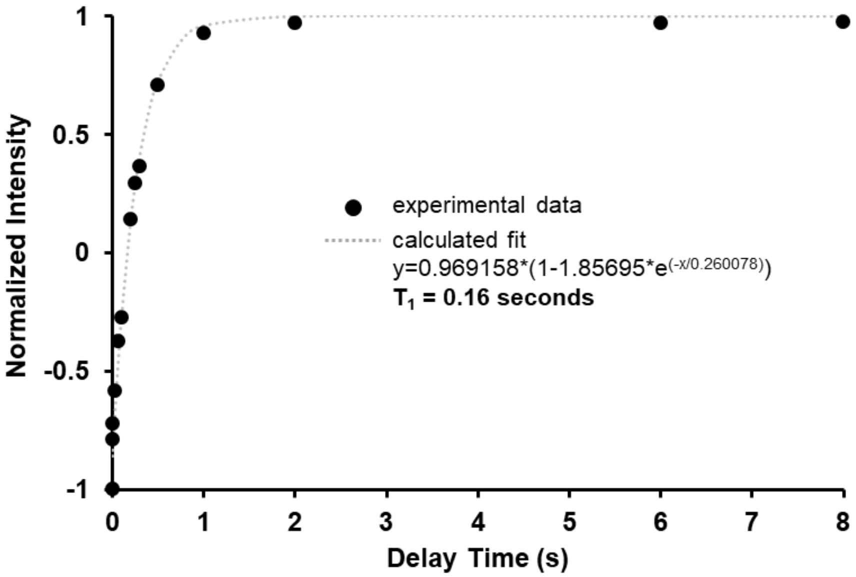

Figure S2. Experimental data from Table S1 (black dots) and the fit (grey dotted line) calculated from the data using LabPlot software.

Table S2. Parameters and error information for the fit generated from the experimental data in Table S1 used to calculate the ^{31}P T_1 time for **6** adsorbed on AC with 98% surface coverage.

Equation: $y = \mathbf{A}\cdot(1-\mathbf{B}\cdot e^{-x/\mathbf{C}})$							
Variable	Value	Uncertainty	Uncertainty (%)	t statistic	P > |t|	Upper	Lower
A	0.969158	0.0320581	3.31	30.2	0	0.898598	1.03972
B	1.85695	0.0452095	2.43	41.1	0	1.75744	1.95645
C	0.260078	0.0164651	6.33	15.8	$6.61\cdot e^{-9}$	0.223838	0.296317

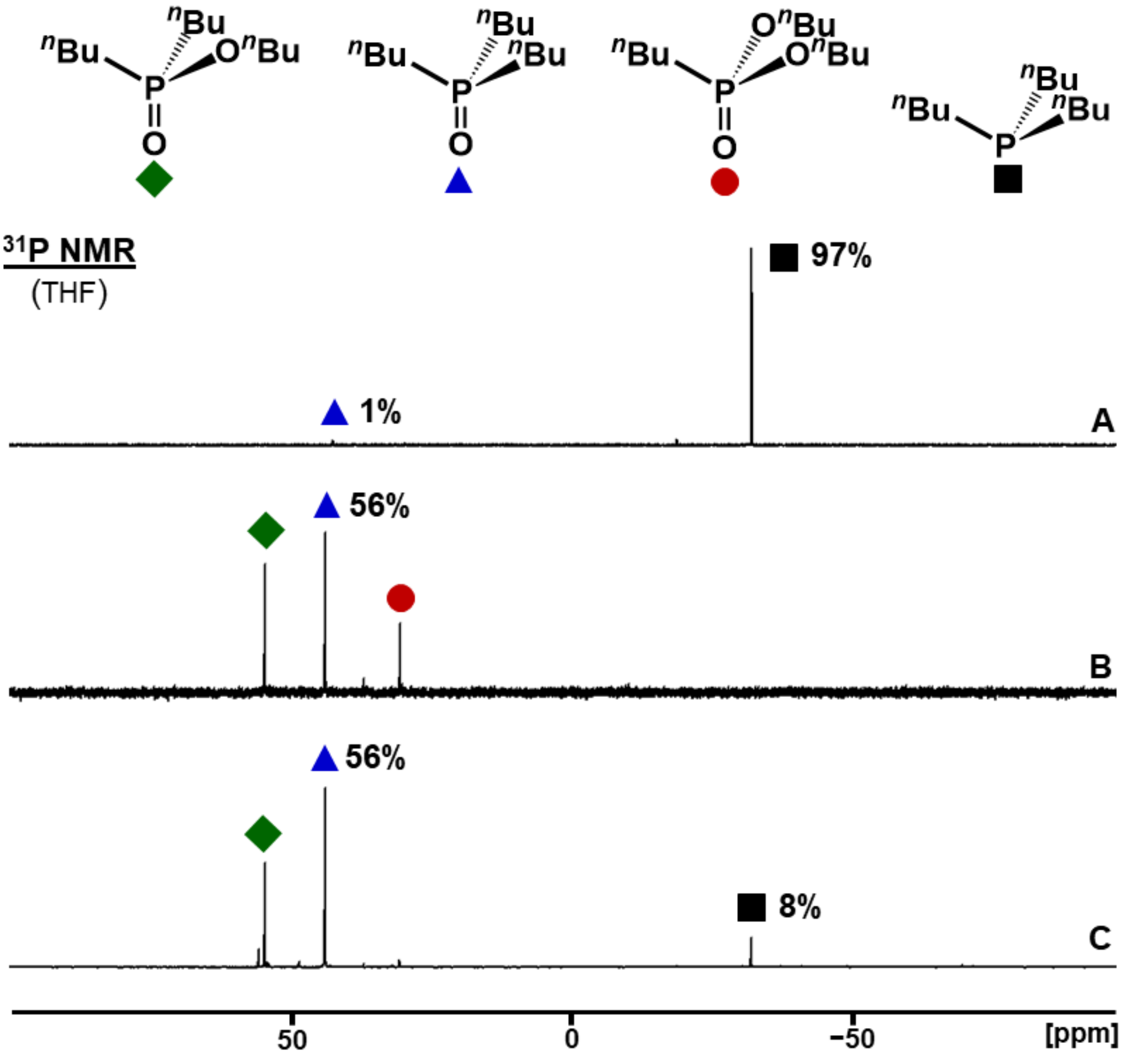

Figure S3. $^{31}P\{^1H\}$ NMR spectra of P^nBu_3 (**1**) prior to oxidation (**A**), oxidized as neat substance in air for 30 minutes (**B**), and oxidized for 30 minutes in air while dissolved in THF (**C**). The given yields of $^nBu_3PO$ were determined by integration. The remaining percentages for each spectrum are accounted for by side products (**A**: 2%, **B**: 44%, **C**: 36%).

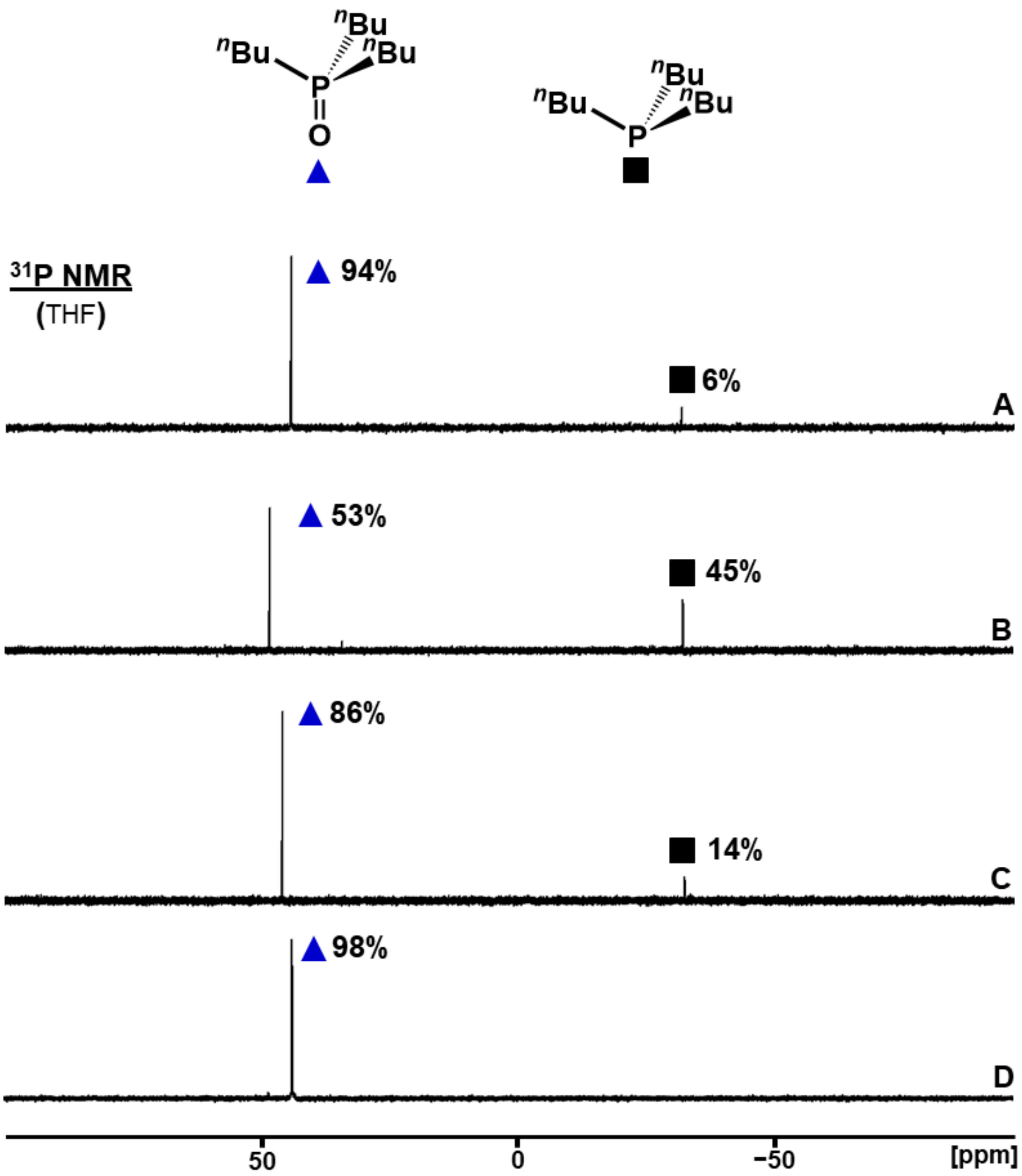

Figure S4. ^{31}P NMR spectra of AC suspensions after various oxidation experiments using **1** adsorbed on AC (Table 1, entries **A-D**). Spectrum **A**: **1** self-adsorbed on AC (150% surface coverage) without solvent overnight and then oxidized in air for 4 hours. Spectrum **B**: **1** adsorbed on AC (150% surface coverage) from THF solution for 20 minutes and then oxidized in air for 1 hour without removing the solvent. Spectrum **C**: **1** adsorbed on AC (150% surface coverage) from THF solution for 20 minutes and then oxidized in air for 1 hour after removing THF *in vacuo*. Spectrum **D**: **1** adsorbed on AC (500% surface coverage) from THF solution for 20 minutes and then oxidized in air for 18.5 hours after removing THF *in vacuo*. Variations in chemical shifts of the signal of $\mathbf{1_{ox}}$ are likely due to slightly different concentrations of the phosphine oxide across the different samples, but all values are within the range of reported values (43-49 ppm).

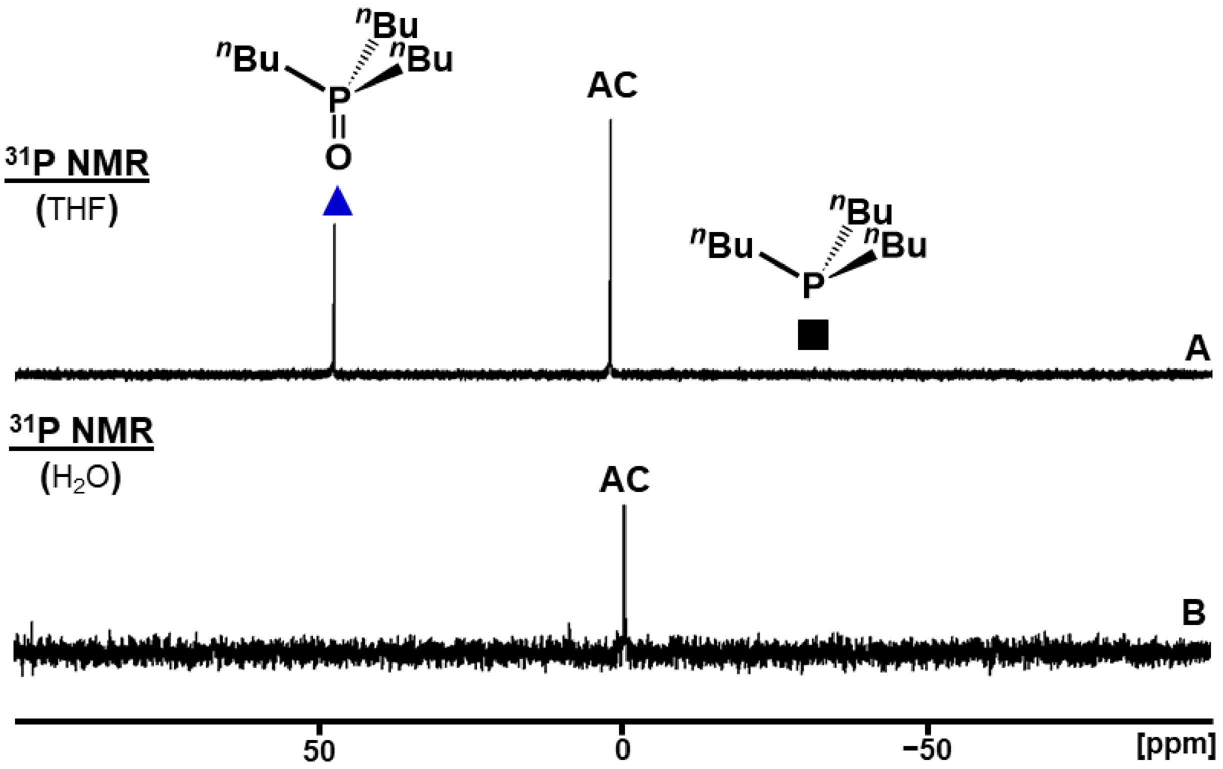

Figure S5. ^{31}P NMR of a suspension of AC after the oxidation of **1**, adsorbed and oxidized solvent-free (**A**) and ^{31}P NMR of the aqueous phase after washing pristine AC with water (**B**).

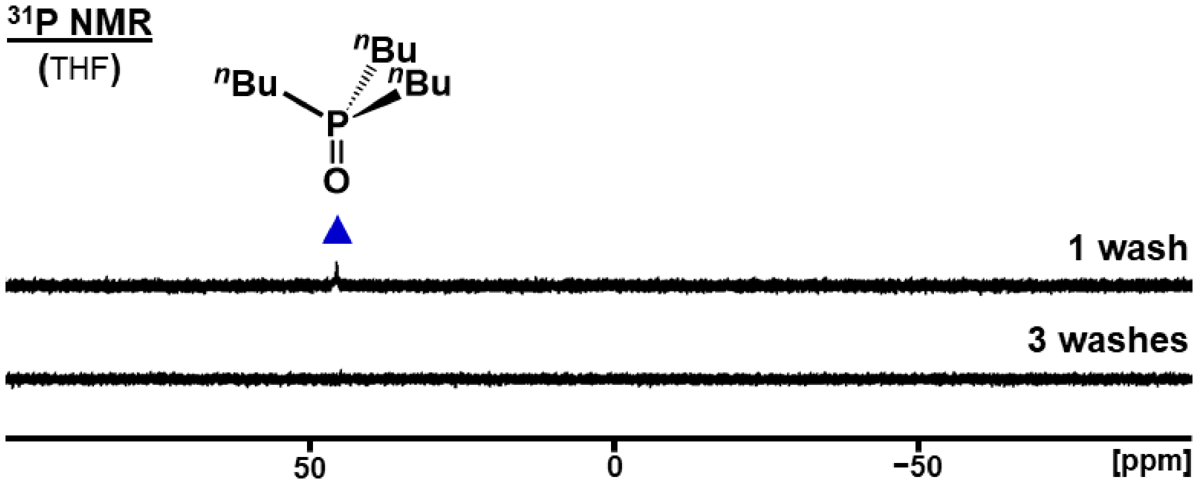

Figure S6. ^{31}P NMR of AC with adsorbed $\mathbf{1_{ox}}$ after one and three THF washings, demonstrating the complete removal of $\mathbf{1_{ox}}$.

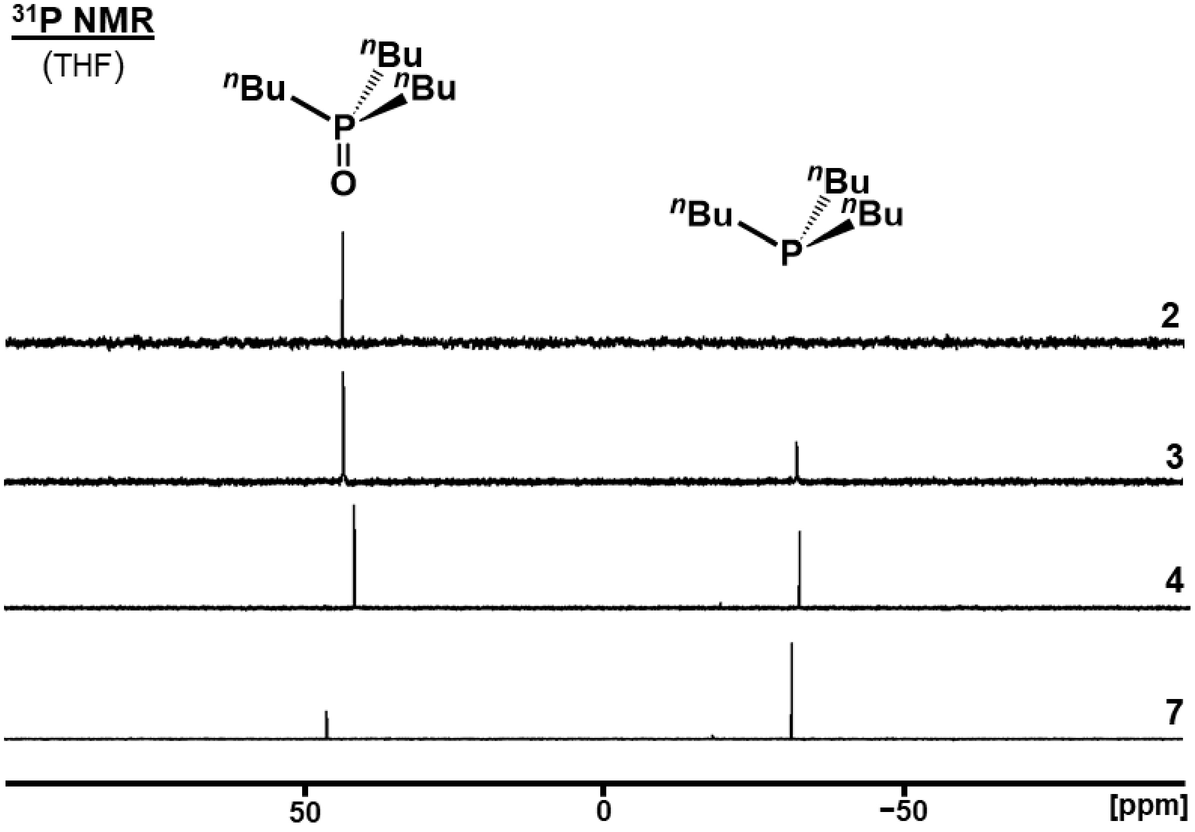

Figure S7. ^{31}P NMR spectra after cycles 2, 3, 4, and 7 (top to bottom) of the oxidation of **1**, reusing the same batch of AC.

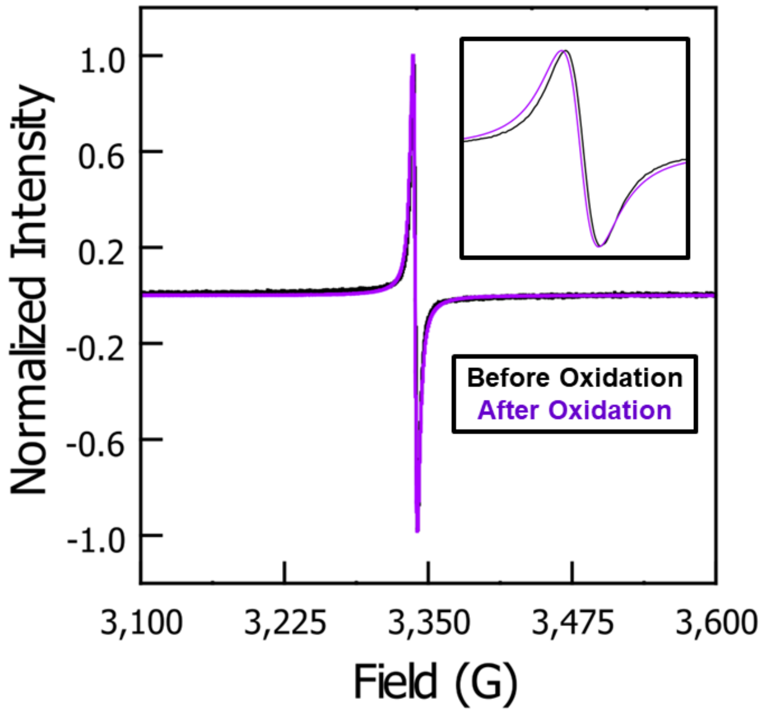

Figure S8. EPR spectra of washed and dried AC prior to any phosphine adsorption and oxidation experiments and the same batch of AC after seven adsorption and oxidation cycles of **1**.

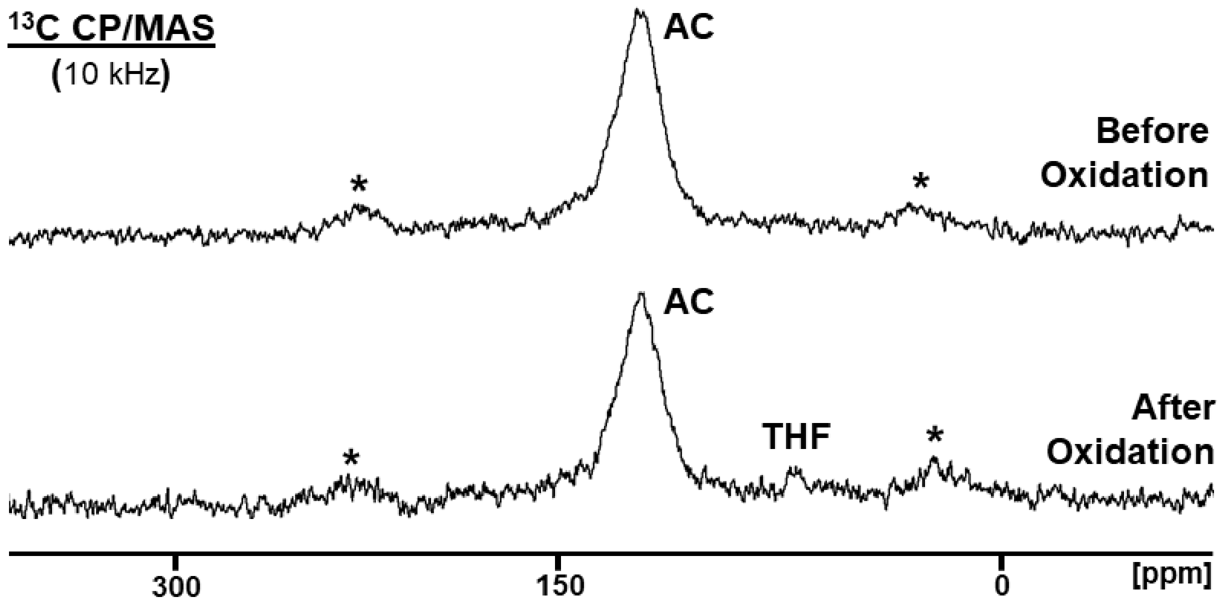

Figure S9. ^{13}C CP/MAS NMR spectra of AC prior to phosphine adsorption (top) and the same AC batch after seven cycles of phosphine adsorption and oxidation (Table 2) (bottom). Asterisks denote rotational sidebands. In the spectrum of the post-oxidation material the peak at about 75 ppm is assigned to the O*C*H_2 carbon of THF. The OCH_2*C*H_2 signal of THF is overlapping with the first order rotational sideband of the AC peak.

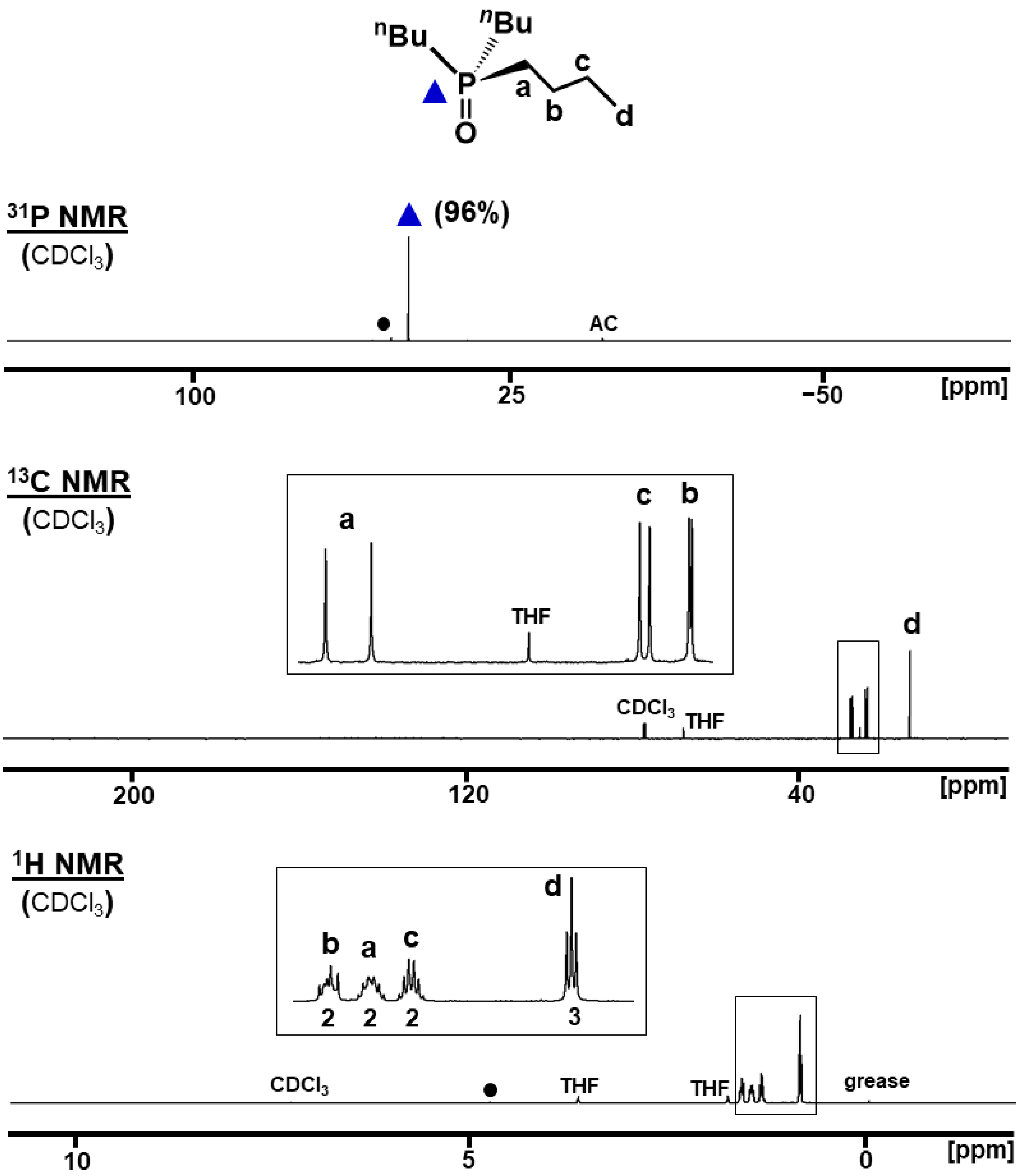

Figure S10. ^{31}P, ^{13}C, and ^{1}H NMR spectra of $\mathbf{1}_{ox}$ obtained by surface-assisted oxidation of **1** adsorbed on AC and washed off with THF. The data match those reported in the literature.[36] Dots denote peaks of side products (4%) and the label AC in the ^{31}P NMR spectrum indicates residual phosphate washed off from the AC along with $\mathbf{1}_{ox}$.

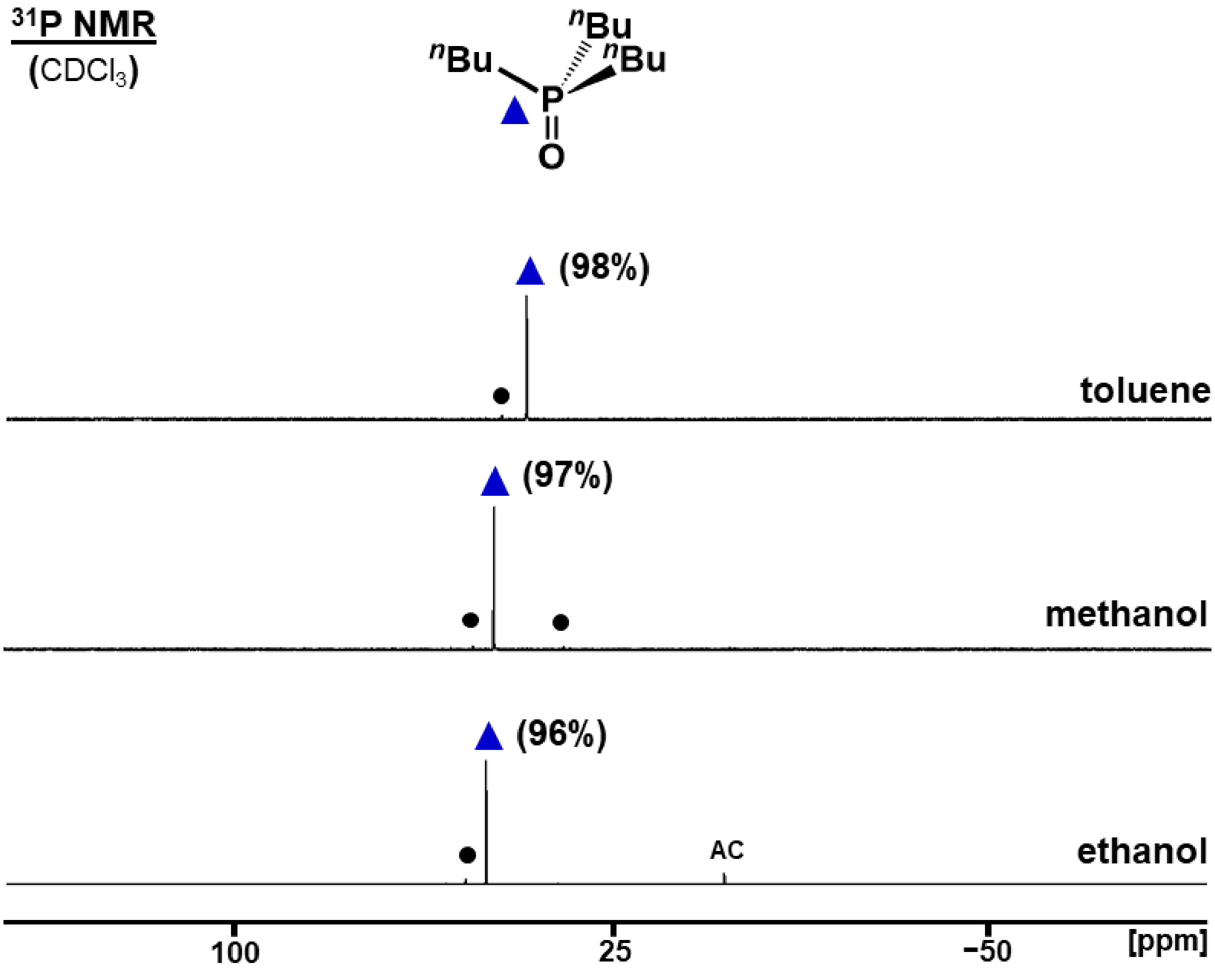

Figure S11. ^{31}P NMR spectra of $\mathbf{1}_{\mathbf{ox}}$ after oxidizing **1** on AC and washing it off with various solvents. Dots denote peaks corresponding to side products (4%) and the label AC in the ^{31}P NMR spectrum indicates residual phosphate washed off from the AC along with $\mathbf{1}_{\mathbf{ox}}$. Variations in the chemical shifts of the signal of $\mathbf{1}_{\mathbf{ox}}$ are likely due to slightly different concentrations across the different samples, but all values are within the range of reported values in various solvents (43-49 ppm).

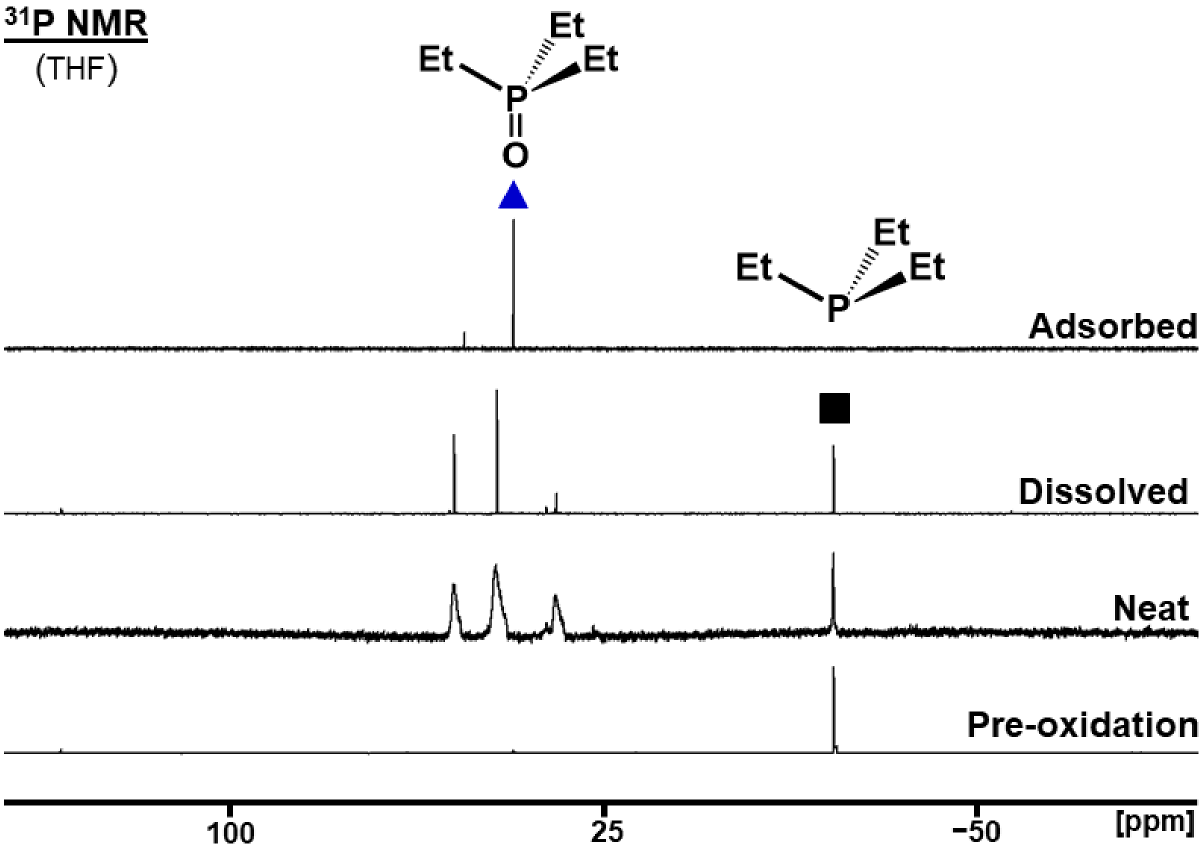

Figure S12. ^{31}P NMR spectra of **2** prior to oxidation (bottom) and after oxidizing the neat phosphine, and **2** dissolved in THF. The top spectrum was recorded after **2** was adsorbed on AC and exposed to the atmosphere for 30 minutes. Yields are reported in Table 4.

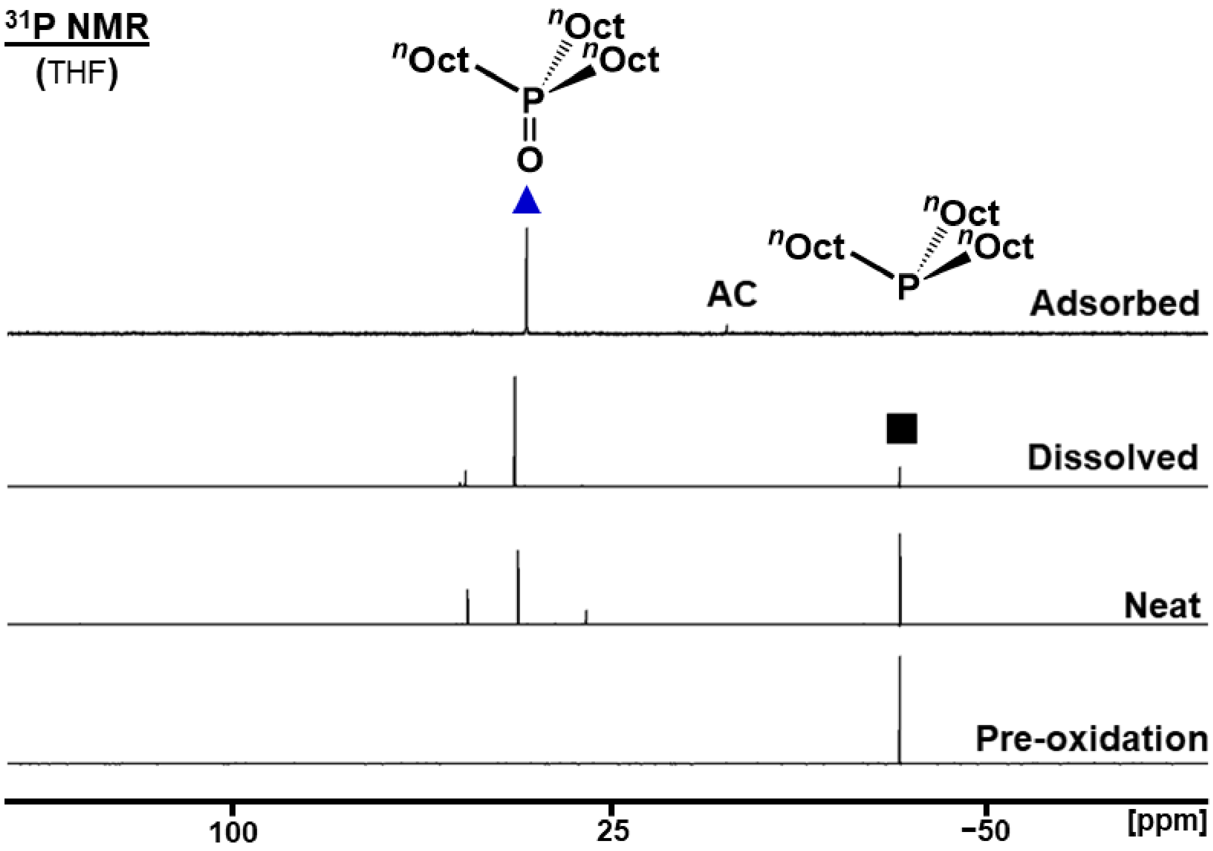

Figure S13. ^{31}P NMR spectra of **3** prior to oxidation (bottom) and after oxidizing the neat phosphine, and **3** dissolved in THF. The top spectrum was recorded after **3** was adsorbed on AC and exposed to the atmosphere for 30 minutes. Yields are reported in Table 4.

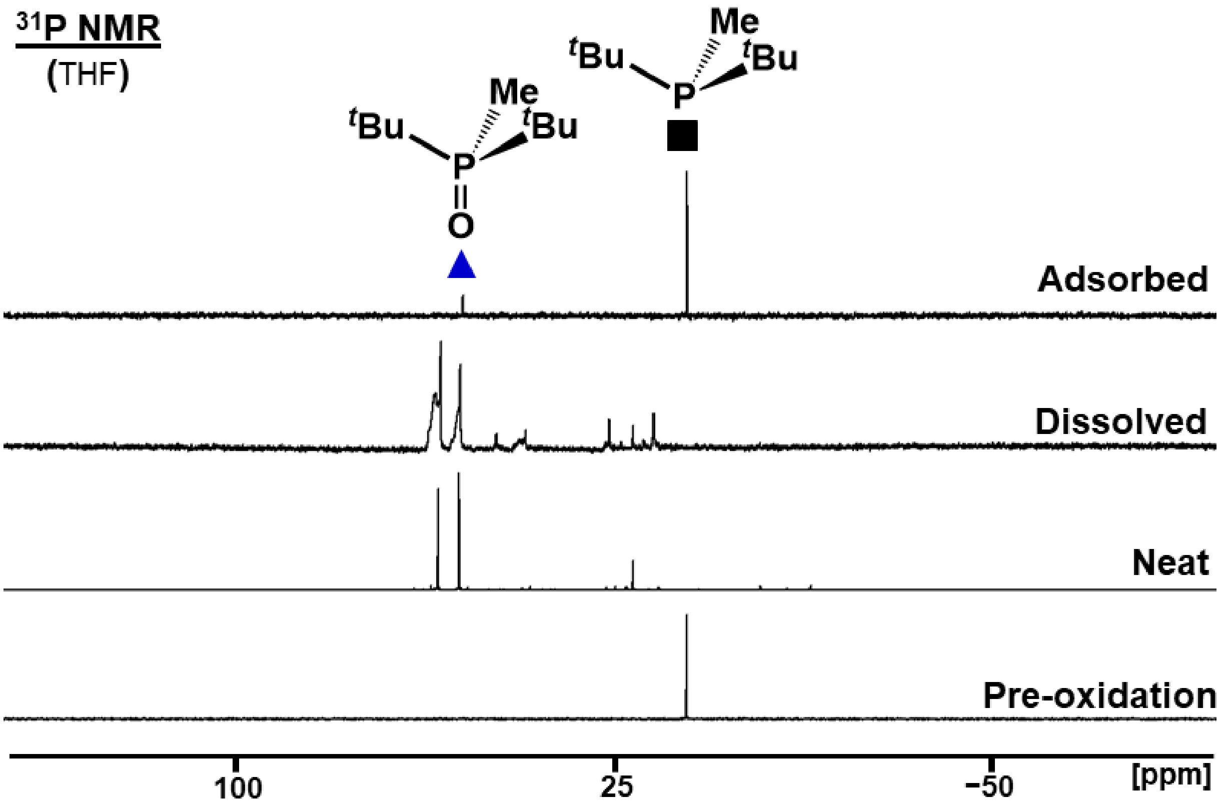

Figure S14. ^{31}P NMR spectra of **4** prior to oxidation (bottom) and after oxidizing the neat phosphine, and **4** dissolved in THF. The top spectrum was recorded after **4** was adsorbed on AC and exposed to the atmosphere for 30 minutes. Yields are reported in Table 4.

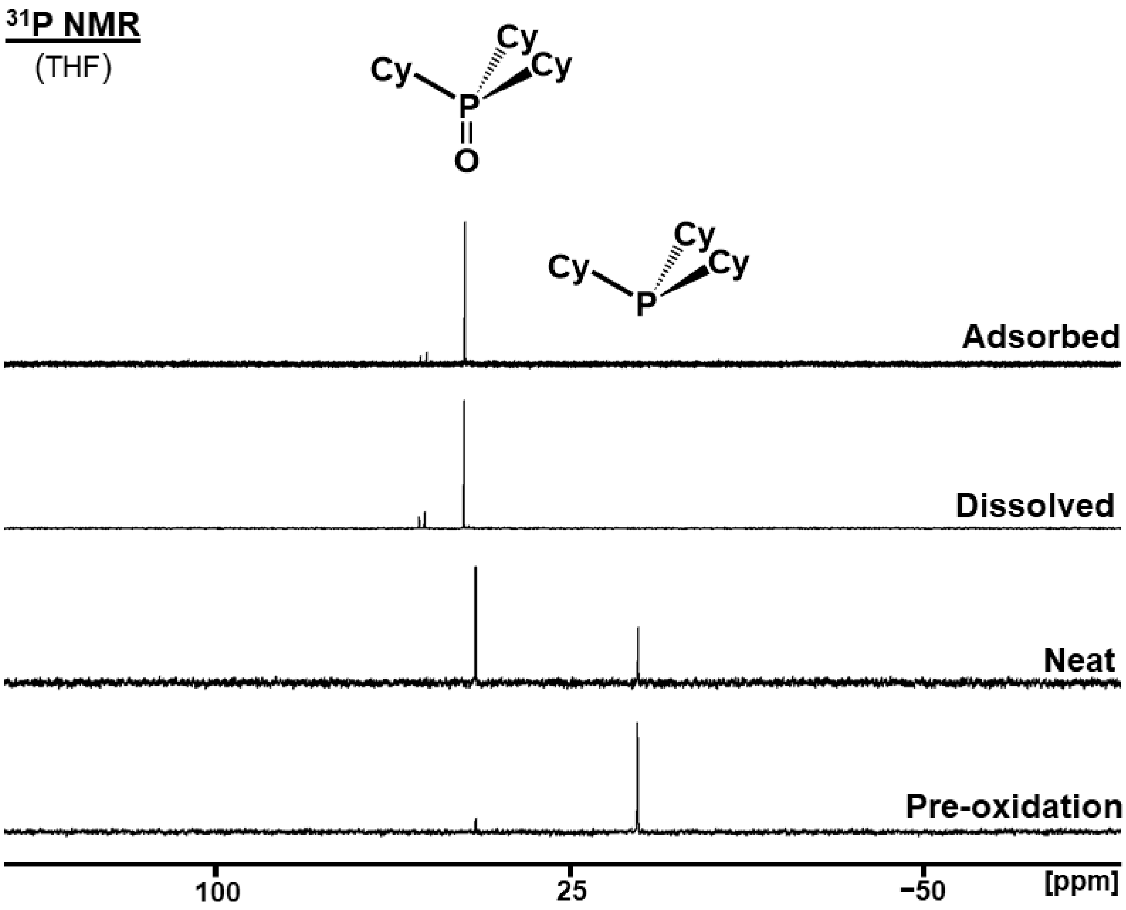

Figure S15. ^{31}P NMR spectra of **5** prior to oxidation and after oxidizing neat **5** and **5** dissolved in THF for 30 minutes. Yields are reported in Table 4.

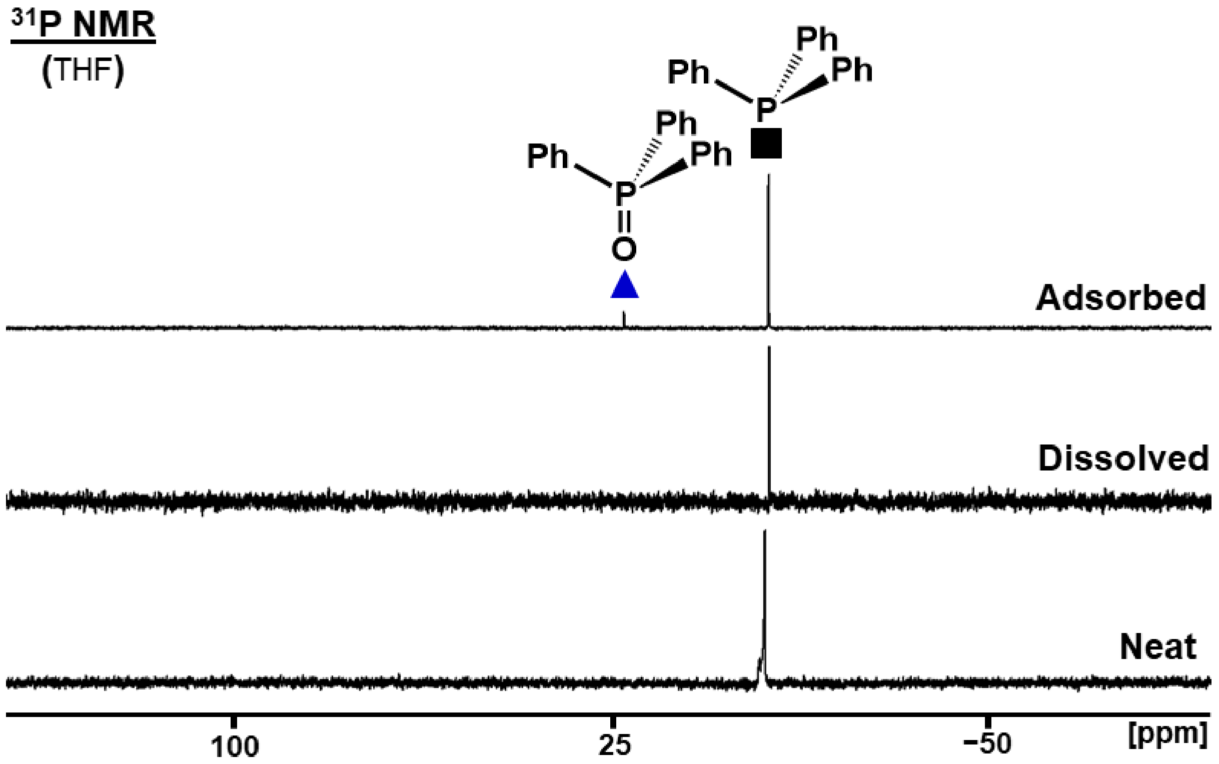

Figure S16. ${}^{31}P$ NMR spectra after exposing neat **6**, its solution in THF, and **6** adsorbed on AC to the atmosphere for 30 minutes. Yields are reported in Table 4.

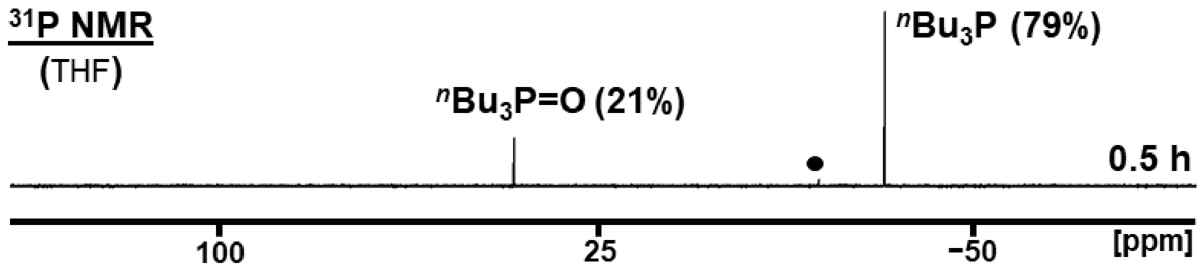

Figure S17. ${}^{31}P$ NMR spectrum of **1** adsorbed and oxidized on the AC brand Norit (81% surface coverage). The dot denotes an impurity in the batch of **1** that was present prior to the oxidation (see also Figure S3, spectrum **A**).

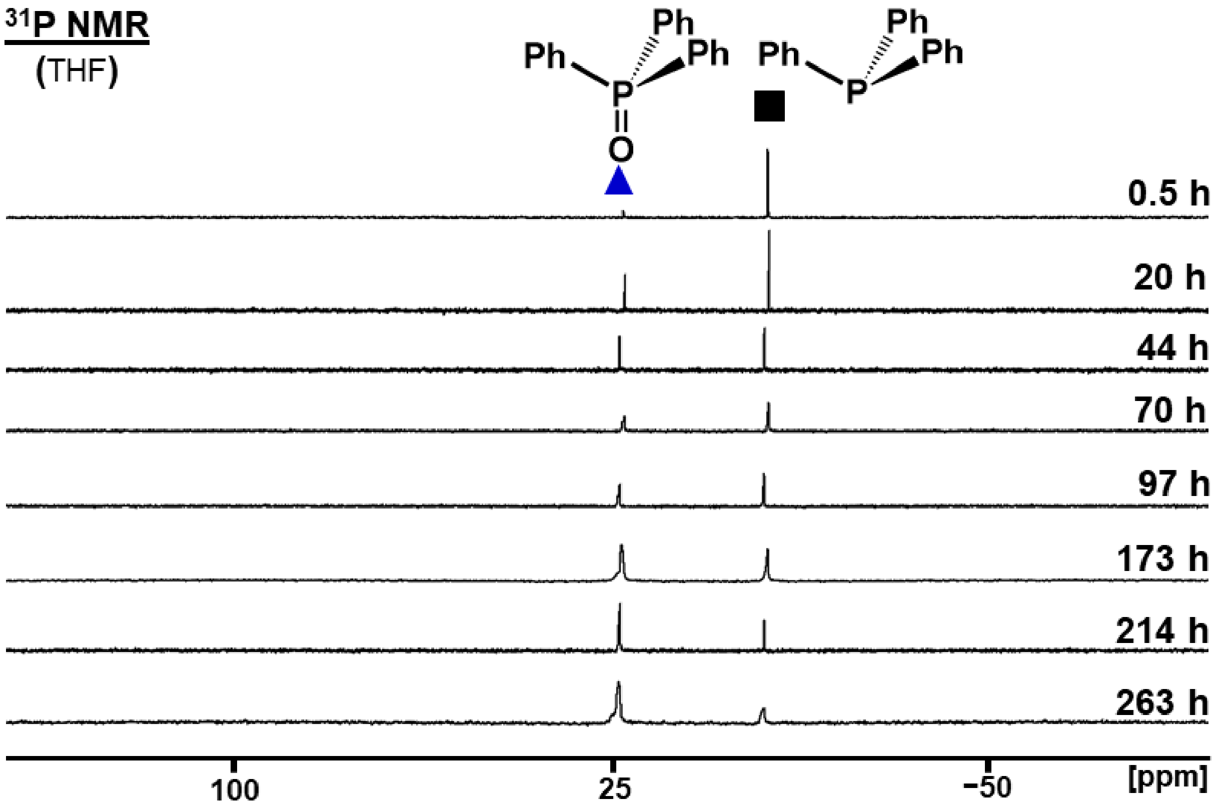

Figure S18. ^{31}P NMR spectra of **6** on AC (98% surface coverage) after exposure to the atmosphere for the indicated times. The yields based on the integration of the signals are reported in Table S1.

Table S3. Oxidation of PPh_3 (**6**) adsorbed on AC with a 98% surface coverage, monitored over time after exposure to the atmosphere. The yields are based on the integration of the NMR signals (Figure S16). The remaining percent of material in the product mixture was residual **6**.

Time (h)	0.5	20	44	70	97	173	214	263
Yield of $\mathbf{6_{ox}}$ (%)	9	31	39	41	40	68	78	78

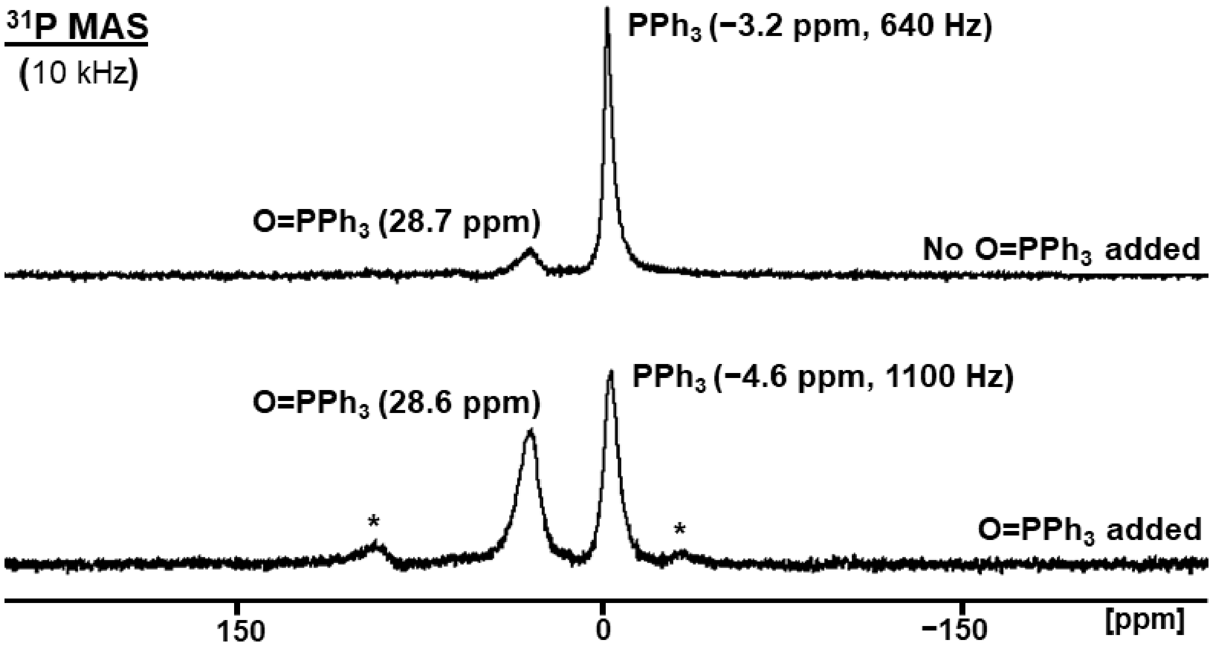

Figure S19. ^{31}P MAS NMR of **6** and $\mathbf{6_{ox}}$ adsorbed on AC for a competition experiment.

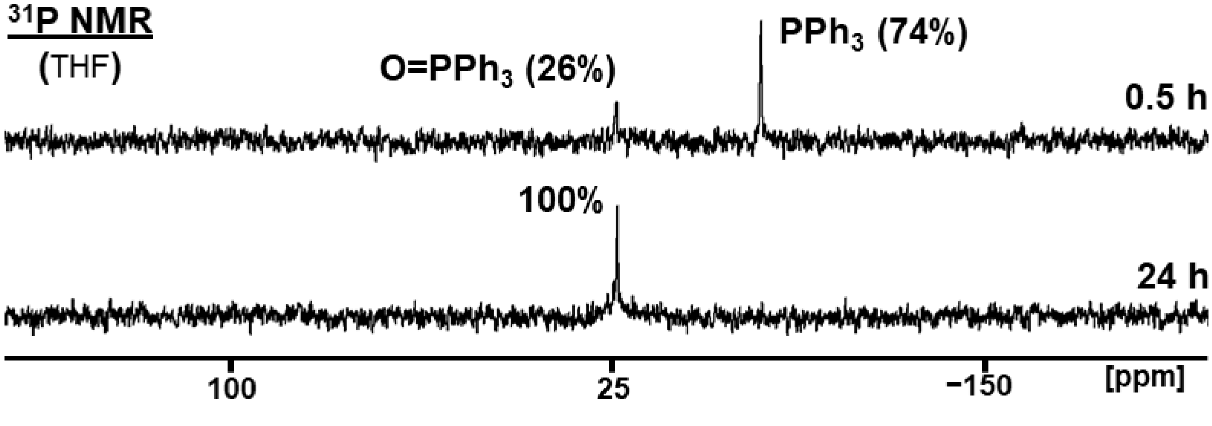

Figure S20. ^{31}P NMR spectra of **6** adsorbed on AC with a 50% surface coverage, measured after 0.5 h and 24 h of exposing the dry material to the atmosphere.

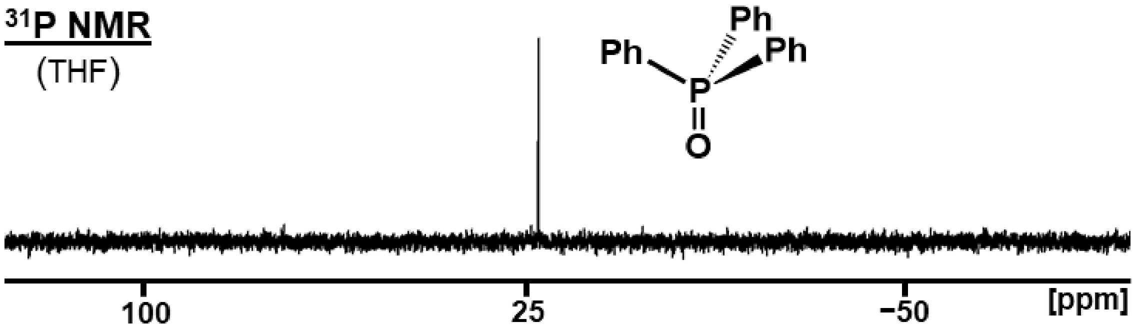

Figure S21. ^{31}P NMR for **6$_{ox}$**, obtained in 92% yield after adsorbing and oxidizing **6** adsorbed on AC in 40% surface coverage for 24 h.

Table S4. Estimated monolayer surface coverages used to calculate approximate surface coverages. Energy minimized models were created and measured using Avogadro software and the monolayer ratios were calculated from the molecular radii (half of the distance between the atoms furthest apart in the energy minimized model with the P atom at the center) and the surface area of DARCO activated cabron (650 m^2/g).

Phosphine	Monolayer Surface Coverage (g/g AC)	Monolayer Surface Coverage (mmol/g AC)
**1**	0.26	1.3
**2**	0.32	2.7
**3**	0.20	0.54
**4**	0.39	2.4
**5**	0.39	1.4
**6**	0.44	1.7

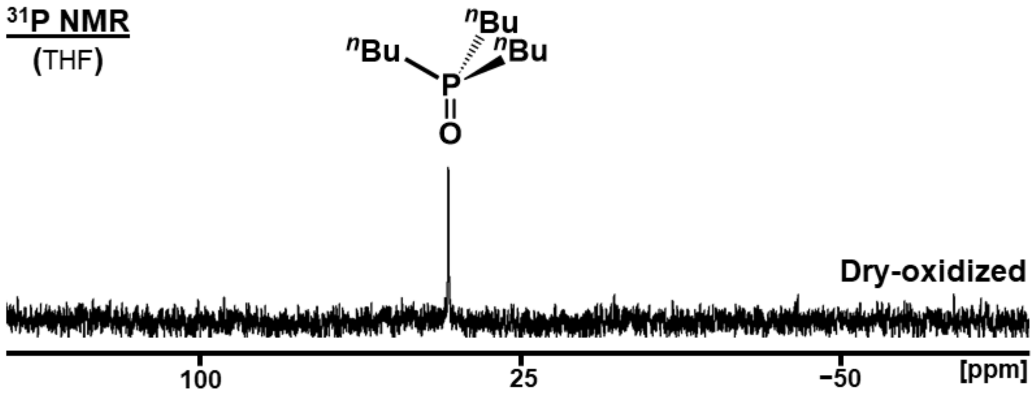

Figure S22. $^{31}P\{^1H\}$ NMR spectrum of O=P^{n}Bu$_3$ (**1**$_{\mathbf{ox}}$) obtained after oxidizing **1**, adsorbed on AC, with dry air.

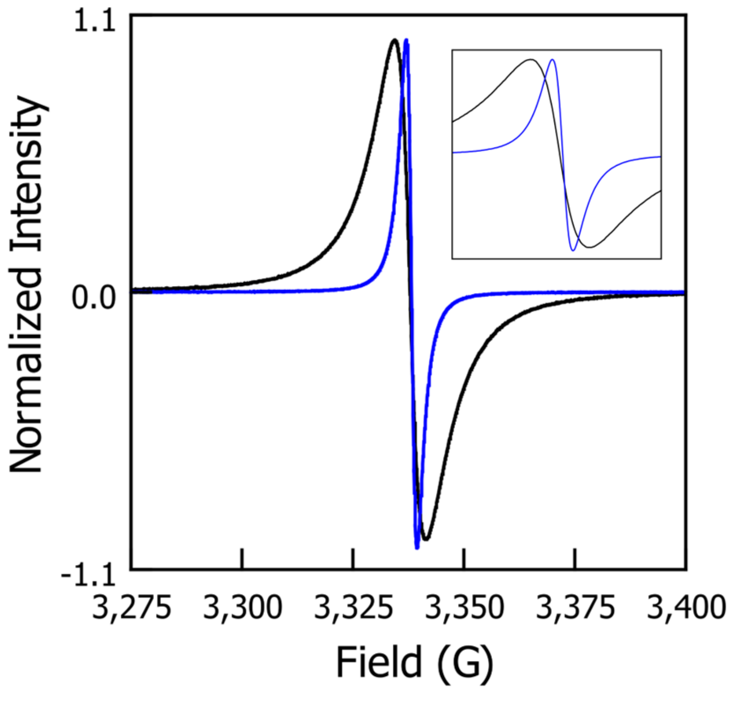

Figure S23. EPR spectra of washed and dried AC under air (blue, 3337 G) and under a nitrogen atmosphere (black, 3334 G).
